# Trial of Selective Early Treatment of Patent Ductus Arteriosus with Ibuprofen

**DOI:** 10.1056/NEJMoa2305582

**Published:** 2024-01-25

**Authors:** Samir Gupta, Nimish V. Subhedar, Jennifer L. Bell, David Field, Ursula Bowler, Elizabeth Hutchison, Sam Johnson, Wilf Kelsall, Justine Pepperell, Tracy Roberts, Sunil Sinha, Kayleigh Stanbury, Jonathan Wyllie, Pollyanna Hardy, Edmund Juszczak, on behalf of the Baby-OSCAR Collaborative Group.

**Affiliations:** 1 Division of Neonatology, Sidra Medicine, Doha, Qatar; 2 Department of Engineering, Durham University, UK; 3 Liverpool Women’s NHS Foundation Trust, Liverpool, UK; 4 National Perinatal Epidemiology Unit Clinical Trials Unit, Nuffield Department of Population Health, University of Oxford, UK; 5 The University of Leicester, Department of Health Science, University Road, George Davies Centre, Leicester, UK; 6 The University of Leicester, Department of Health Science, University Road, George Davies Centre, Leicester, UK; 7 NICU, Rosie Hospital, Cambridge University Hospital Foundation Trust, Cambridge, UK; 8 Institute of Applied Health Research, University of Birmingham, Birmingham, UK; 9 South Tees Hospitals NHS Foundation Trust, James Cook University Hospital, Middlesbrough, UK; 10 School of Medicine, University of Nottingham, Nottingham, UK

## Abstract

**Background:**

The cyclooxygenase inhibitor ibuprofen may be used to treat a patent ductus arteriosus (PDA) in preterm infants. We hypothesized that selective early treatment of large PDAs with ibuprofen would improve short-term outcomes.

**Methods:**

In this multicenter, randomized, double-blind, placebo-controlled trial evaluating early (up to 72 hours) treatment with ibuprofen for a large PDA (diameter □ 1.5 mm and pulsatile flow) in extreme preterm infants (born between 23 weeks+0 days and 28 weeks+6 days’ gestation), we randomly assigned 326 infants to receive ibuprofen and 327 to receive placebo. The primary outcome was a composite of death or moderate or severe bronchopulmonary dysplasia (BPD) at 36 weeks post-menstrual age.

**Results:**

The primary outcome occurred in 220 of 318 infants (69.2%) assigned to ibuprofen and 202 of 318 infants (63.5%) assigned to placebo (adjusted risk ratio [aRR], 1.09; 95% confidence interval (CI), 0.98 to 1.20; p=0.10). Death occurred in 44 of 323 infants (13.6%) assigned to ibuprofen and 33 of 321 infants (10.3%) assigned to placebo (aRR, 1.32; 95% CI, 0.92 to 1.90). Among infants that survived to 36 weeks’ post-menstrual age, moderate or severe BPD occurred in 176 of 274 infants (64.2%) assigned to ibuprofen and 169 of 285 infants (59.3%) assigned to placebo (aRR 1.09, 95% CI 0.96 to 1.23). There were two severe adverse events that were possibly related to ibuprofen.

**Conclusions:**

Early treatment with ibuprofen was not associated with an improvement in the composite outcome of death or moderate or severe BPD at 36 weeks’ post-menstrual age compared to placebo. (Funded by the National Institute for Health Research (NIHR) Health Technology Assessment programme; Baby-OSCAR ISRCTN Registry number, ISRCTN84264977)

Over the last two decades, the survival of extreme preterm infants has increased with modest reductions in neonatal morbidities, although the incidence of bronchopulmonary dysplasia (BPD) has increased.^1^ In this gestation group, a large patent ductus arteriosus (PDA) (1.5 mm diameter or greater) that is present beyond 3 days of age is associated with an increase in the odds of death or severe morbidity and BPD compared to infants without a PDA.^2^ The incidence of PDA is inversely proportional to gestational age: over 40% of infants born at less than 28 weeks’ gestation have a persistent PDA by 4 months of age.^3–4^ The risk of BPD or death in extreme preterm infants also increases with persistence of the PDA beyond 1–2 weeks of age.^5^

Various treatment strategies have been investigated for managing infants with a PDA.^6^ Prophylaxis with indomethacin or ibuprofen in the first 12 to 24 hours of life is reported to reduce severe intraventricular hemorrhage and pulmonary hemorrhage, but without an improvement in survival without neurosensory impairment at 18 months.^7^ However, utilizing a prophylactic approach, most infants will receive treatment unnecessarily since PDAs can close spontaneously.^8^ Data are limited on the treatment of infants with a symptomatic PDA, with no reported improvement in clinical outcomes.^9^ Consequently, the number of extreme preterm infants with a PDA that are treated pharmacologically has decreased.^10^

By utilizing bedside functional echocardiography,^11^ infants can now be screened to identify PDAs that are large with unrestricted flow that are unlikely to close spontaneously. Selective early targeted treatment of patients with these PDAs may avoid unnecessary treatment of all patients with PDAs.^12^ Parenteral indomethacin and ibuprofen have been used for early targeted treatment of PDA with no evidence of a difference in their efficacy,^13^ although the side-effect profile of ibuprofen is reported to be superior.^6^

We hypothesized that early (up to 72 hours) selective treatment of patients who have a PDA that is 1.5 mm diameter or larger with unrestricted flow, identified using bedside echocardiography, with ibuprofen compared to placebo, would reduce mortality and improve short-term outcomes such as BPD.

## Methods

### Trial Design

A multicenter, randomized, double-blind, placebo-controlled trial was conducted in 32 neonatal intensive care units in the United Kingdom following a published protocol^14^ (available online with the full text of this article at NEJM.org). Treatment assignment was blinded from the clinicians, the infant’s family and individuals who assessed outcomes.^14^ The trial was coordinated by the NPEU Clinical Trials Unit at the University of Oxford, United Kingdom (the trial sponsor), and overseen by the Trial Steering Committee, acting on the recommendations of an independent Data Monitoring Committee. Additional details are available in the Supplementary Appendix available online with the full text of this article at NEJM.org.

### Patients

After written informed consent was obtained from parents, infants born between 23 weeks+0 days to 28 weeks+6 days’ gestation, less than 72 hours old and confirmed by echocardiography to have a large PDA, were considered eligible. A large PDA was defined as ≥1.5mm in diameter with unrestricted transductal pulsatile (left-to-right shunting) flow and no clinical concerns for acute pulmonary hypertension. A full list of inclusion and exclusion criteria are shown in Table S1 in the Supplementary Appendix.

### Randomization

Dynamic assignment to treatment group was performed via a secure web-based randomization system that was created and hosted by the NPEU Clinical Trials Unit with 24/7 telephone back-up, ensuring concealment of group assignment. The randomization program used a probabilistic minimization algorithm and assigned patients to groups in a 1:1 ratio to ensure balance between the groups for the size of the PDA, gestational age at birth, age, sex, site, multiple births, mode of respiratory support and receiving inotropes. Multiple births were randomized individually. Participants were enrolled by the delegated clinician at the study site.^14^

### Intervention and Study Procedures

The trial intervention as ibuprofen sodium and referred to as the investigational medicinal product (IMP). The matched placebo was supplied as a clear sterile solution of 0.9% sodium chloride. Each carton was labelled with a unique code in compliance with the guidance given in Annex 13 of the European Commission’s guidelines for Good Manufacturing Practice. Ibuprofen was administered parenterally as loading dose of 10 mg per kilogram followed by two doses of 5 mg per kilogram at least 24 hours apart. Placebo was administered as equal volume of 0.9% sodium chloride (Table S2). Only one course of trial intervention was given, and infants were screened with echocardiography at 3 weeks of age with the intention of assessing ductal patency while minimizing open-label treatment. Predefined criteria for open-label medical or surgical treatment after enrollment are shown in Table S3.

Transthoracic echocardiography was performed to assess eligibility within 72 hours of birth and at 3 weeks postnatal age (18 to 24 days) to assess the patency of the PDA. For quality control, a sample of echocardiograms from 65 infants was reviewed independently by a pediatric echocardiographer, blinded to treatment allocation.

### Outcomes

The primary outcome was a composite of death or moderate or severe BPD at 36 weeks post-menstrual age^15^ (Tables S4 and S5). A physiologic challenge of supplemental oxygen reduction was used to test for oxygen need at 36 weeks post-menstrual age^16^ to differentiate mild from moderate BPD (Fig. S1). Secondary short-term outcomes up to the time of discharge included individual components of the primary outcome, severity of BPD, severe intraventricular hemorrhage, cystic periventricular leukomalacia, retinopathy of prematurity requiring treatment, significant pulmonary hemorrhage, acute pulmonary hypertension, definitive necrotizing enterocolitis, closed or non-significant PDA <1.5 mm with restricted flow at 3 weeks age, open-label treatment of a PDA causing symptoms, weight gain and discharge home on oxygen (Table S5).^14^ Other secondary short-term outcomes are listed in the Supplementary Appendix and the Statistical Analysis Plan available online at NEJM.org.^17^

All outcome data were recorded routinely, including demographic data and complications of prematurity, which were obtained from clinical notes or trial-related assessments (Table S6).

### Statistical Analysis

The incidence of the primary outcome was estimated to be 60% (Supplementary Methods). A sample size of 730 infants would be required to detect a clinically important absolute risk reduction of 12% with 90% power and a type I error of 5% from a control group event rate of 60% to a treatment group event rate of 48%, assuming 1% of infants were lost to follow-up.^14^ Analyses were performed according to the intention-to-treat principle, excluding infants from the analysis only if their data were missing. Missing data were not imputed. Analyses were adjusted for minimization factors, such as the size of the PDA at randomization, gestational age at birth, age at randomization, sex, multiple birth, mode of respiratory support at randomization, receiving inotropes at time of randomization, and center, and the correlation between siblings from multiple births, where technically possible (Table S7). Binary outcomes were analyzed using mixed effects Poisson regression with a robust variance estimator with risk ratios and 95% confidence intervals presented. Model diagnostics were checked and satisfied (Fig. S2). Continuous outcomes were analysed using linear regression models, with mean differences and 95% confidence intervals presented, after checking model assumptions. Due to the large number of short-term outcomes, statistical inference was restricted to a predefined shortlist (Table S5). No formal method to adjust for multiplicity was used; the widths of the confidence intervals have not been adjusted for multiplicity and inferences drawn may not be reproducible and should not be used to infer definitive treatment effects for secondary outcomes. Full details of the statistical analysis are documented in the Statistical Analysis Plan.^17^ Additional details are presented in the Supplementary Appendix. The statistical software Stata/SE version 15 was used for all analyses.

## Results

### Patients

**Figure 1:**
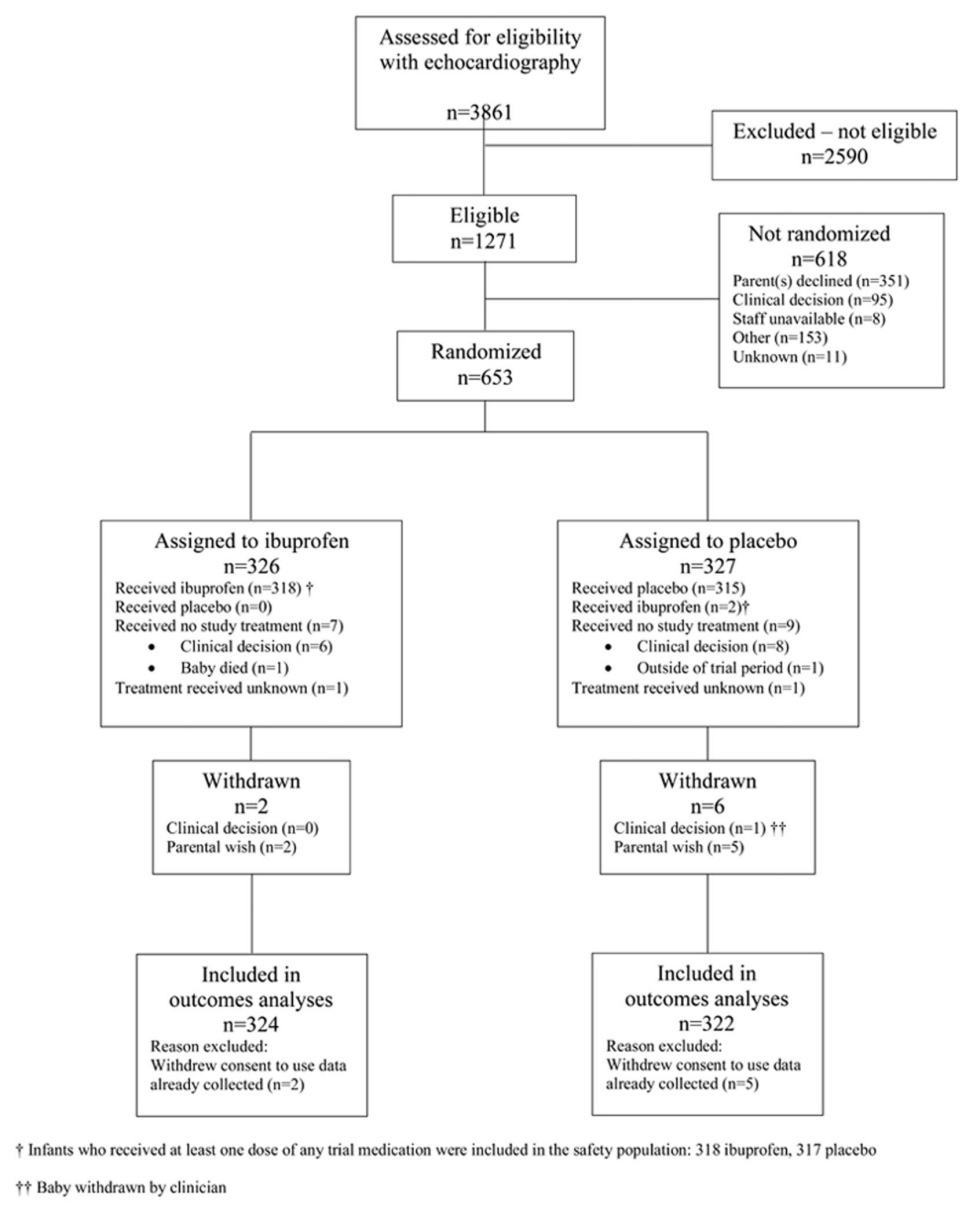
Participant flow

Between July 2015 and December 2020, a total of 653 infants were randomized: 326 patients were assigned to receive ibuprofen and 327 patients were assigned to placebo. A total of 22 patients were randomized during the internal pilot phase and 631 during the main recruitment phase (Figure 1 and Table S9). Maternal and infant baseline characteristics appeared well balanced between the groups (Table 1). The median diameter of the PDA was 2.2 mm (interquartile range, 1.9 to 2.6). An independent audit of the echocardiograms indicated that 93.8% of infants randomized in the trial met the pre-defined echocardiography eligibility criteria. The remainder of the echocardiographic images could not be assessed accurately to confirm the eligibility criteria.

A total 318 of 326 infants (97.5%) assigned to ibuprofen and 315 of 327 (96.3%) assigned to placebo received their allocated intervention (Figure 1). Parenteral consent to use the data collected from 7 infants was withdrawn. Data required to assess the primary outcome was missing from an additional 10 infants. Assessment of the primary outcome was possible in 318 infants (97.5%) in the ibuprofen group and 318 (97.2%) in the placebo group (Figure 1).

### Outcomes

The primary outcome of death or moderate or severe BPD at 36 weeks’ post-menstrual age occurred in 220 of 318 infants (69.2%) assigned to ibuprofen compared to 202 of 318 infants (63.5%) assigned to placebo (adjusted risk ratio [aRR], 1.09; 95% CI, 0.98 to 1.20; p=0.10) (Table 2). Death occurred in 44 of 323 infants (13.6%) assigned to ibuprofen and in 33 of 321 (10.3%) assigned to placebo (aRR, 1.32; 95% CI, 0.92 to 1.90). Among those infants who survived to 36 weeks’ post-menstrual age, moderate or severe BPD was present in 176 of 274 infants (64.2%) assigned to ibuprofen and 169 of 285 (59.3%) assigned to placebo (aRR, 1.09; 95% CI, 0.96 to 1.23) (Table 2). Results for the primary outcome excluding infants who received open-label medical treatment without meeting the specified criteria are shown in Table S10.

**Figure 2:**
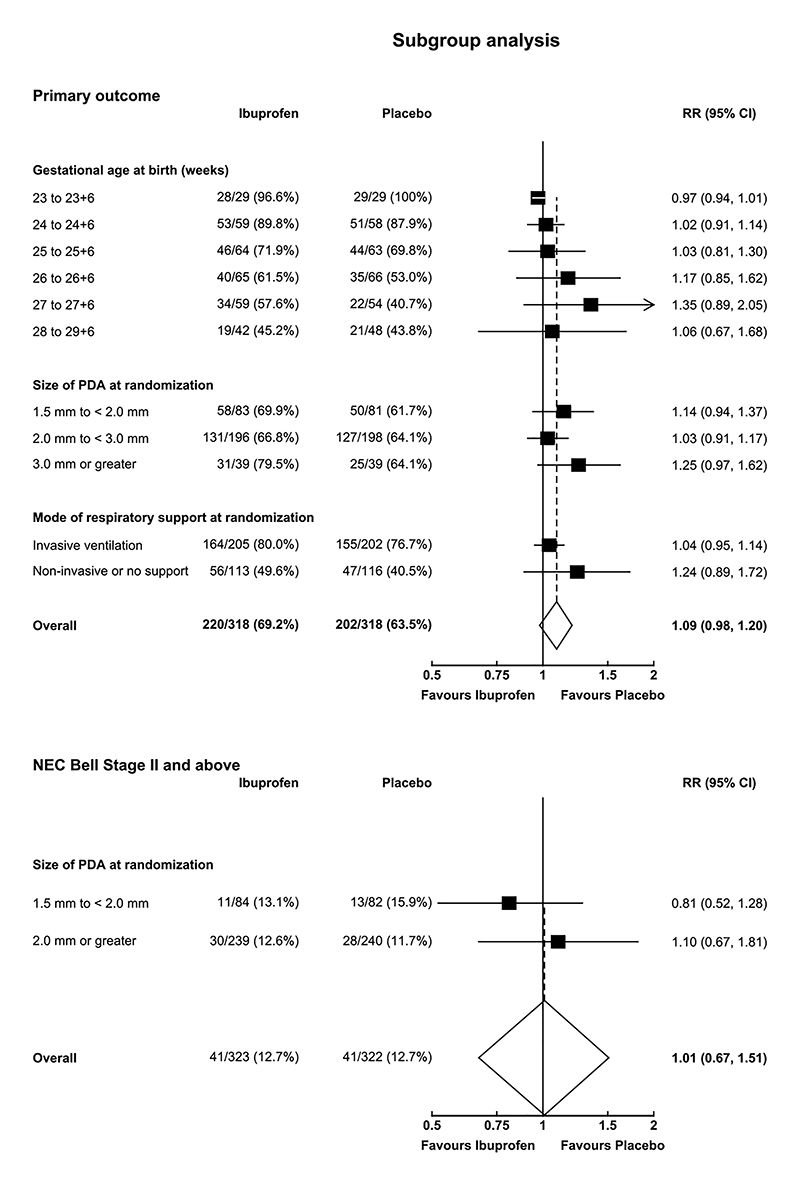
Forest plot of primary outcome and NEC Bell Stage II subgroup analyses Risk ratios and 95% confidence intervals were obtained from an interaction term between treatment assignment and subgroup characteristic of interest, in a log binomial model adjusted for size of PDA at randomization, gestational age at birth, age at randomization, sex, multiple birth, mode of respiratory support at randomization, receiving inotropes at time of randomization, and centre as a random effect, and clustered by siblings to account for correlation between multiple births. Some of the patient subgroups have been collapsed into fewer subgroups than pre-specified due to the low number of patients in each category. No adjustments were made for multiplicity of testing and therefore interpretation of the confidence intervals should not be used to assess treatment effect.

A closed or small PDA (diameter <1.5 mm) at around 3 weeks of age was present in 176 of 317 patients (55.5%) in the ibuprofen group and 117 of 316 patients (37.0%) in the placebo group (aRR 1.50, 95% CI 1.30 to 1.74; Table 2). After randomization 571 of 646 infants (88.4%) received all 3 doses of the assigned treatment (Table 3). A total of 43 infants (13.3%) assigned to ibuprofen and 82 infants (25.5%) in the placebo group required open-label medical treatment for symptoms attributable to a PDA (Table S13); 9 infants (2.8%) assigned to ibuprofen and 31 infants (9.6%) assigned to placebo required surgical treatment (Table 2). The rate of open-label treatment, including surgical ligation, was 14.2% compared to 29.8% at a median time from randomization of 11 days (interquartile range, 8 to 17) compared to 12 days (interquartile range, 7 to 21) among the patients assigned to ibuprofen and placebo, respectively (Tables S12 and S13, Fig. S4). Other secondary outcomes are shown in Table 2 and Table S13. Prespecified subgroup analyses for the composite primary outcome or its components are shown in Figure 2 and Figures S3 and S4. The relationship between the size of the PDA at randomization and severe necrotizing enterocolitis (Bell Stage II and above) is shown in Figure 2.

### Safety

There were two serious adverse events that were assessed as possibly related to ibuprofen. There were 7 unforseeable serious adverse events (5 in the ibuprofen group and 2 in the placebo group); 6 of these events were assessed as not related to ibuprofen and one possibly related to ibuprofen. There was one suspected unexpected serious adverse event in a patient in the ibuprofen group that was assessed as possibly related to ibuprofen. Foreseeable serious adverse events were reportable between the first dose of trial medication and 7 days after the last dose (Tables S15 and S16).

### Discussion

In this randomized double-blind placebo-controlled trial of extreme preterm infants with a large PDA, there was no evidence that early treatment with ibuprofen was associated with an improvement in the composite outcome of death or moderate or severe BPD at 36 weeks’ post-menstrual age. There were no apparent between-group differences in death or moderate or severe BPD.

Our results are broadly consistent with other studies of early targeted treatment of PDA with ibuprofen that have not demonstrated a convincing benefit in clinical outcomes.^18–22^ Although these studies have demonstrated a reduced risk of pulmonary hemorrhage and patient symptoms attributable to the PDA, intervention with ibuprofen is not associated with a reduction in the incidence of BPD, mortality or neurodisability. ^19–20^

Approximately half of the infants we enrolled were less than 26 weeks’ gestation, the cohort at greatest risk of developing a hemodynamically significant PDA. We enrolled infants with a large PDA based on a combination of echocardiographic parameters, including the diameter of the PDA and ductal flow characteristics.^23–24^ The rates of death or moderate or severe BPD in our study were high, but comparable to other randomized trials of early pharmacological treatment of PDA.^19 20 22^

A single early course of ibuprofen resulted in a closed or small PDA (confirmed by echocardiography at 3 weeks of age) in only 55.5% of infants randomized to ibuprofen. Although previous studies have reported variable closure rates with early intravenous ibuprofen therapy,^25^ our findings are consistent with recent studies in similar patient populations.^19 22 26^ Variation in the rate of PDA closure among studies is likely explained by differences in the timing of the intervention, an open-label treatment study design, the route of administration of the ibuprofen and the dosing regimen used.^6 27^ Intravenous ibuprofen using a standard dosing regimen was chosen as the intervention in our study because higher doses are usually only used when an infant is 7 days of age or older, and it was the most common treatment schedule for infants with a PDA in the United Kingdom at the time the study was designed.^28^ Similarly, most units did not routinely repeat echocardiographic assessment after ibuprofen therapy with the intention of offering a second course if the PDA remained patent.

The rate of open-label medical therapy for patients with a symptomatic PDA in this trial appeared lower than that reported in other similar trials^29^ and occurred almost twice as frequently in the placebo group compared to the ibuprofen group. Although a recent non-inferiority trial of early ibuprofen in the management of patients with a PDA achieved a very low rate of open-label medical therapy of 0.7% in the expectant management arm,^20^ there was frequent use of acetaminophen as an analgesic after randomization in both arms.

Accordingly, exposure to any pharmacological agent with the potential for ductal closure was approximately 25% in babies managed expectantly, representing a contamination rate comparable to ours.

The median time from randomization to open-label medical therapy in our study was 11 to 12 days, well-beyond when the infant received the trial intervention (up to 7 days). This was much later than the timing of rescue treatment in a French study^19^ (median 4 days) that overlapped with the trial intervention. The low effective closure rate in our ibuprofen group combined with the relatively late timing of open-label medical therapy suggests that a large proportion of treated infants may have been exposed to the potentially damaging effects of the ductal shunt for a prolonged time period. Combined with the relatively high incidence of open-label medical therapy in the placebo group (30%), this resulted in poor discrimination between our two study groups with respect to ductal patency (55.5% versus 37.0%) and probably also in prolonged exposure to a ductal shunt. The absence of data from serial echocardiograms precludes detailed analysis and limits interpretation of the impact of shunt duration on outcomes between the two groups. Nevertheless, one might reasonably conclude that an early, echo-targeted, course of ibuprofen results in an additional closure or constriction rate of 18% without impacting clinical outcomes.

We found no evidence that ibuprofen resulted in excess serious complications. Unlike Hundscheid *et al*, we did not identify an association between ibuprofen therapy and BPD, a finding that might be explained by differences in study populations or drug exposure.^20^ Whereas most infants enrolled into our study were receiving invasive ventilation, the Beneductus trial mostly enrolled those who were on non-invasive respiratory support. Another important difference was the use of repeated courses (often with high doses) of ibuprofen in the Beneductus trial. This early exposure to high cumulative doses of ibuprofen in infants with a relatively low baseline risk of BPD as in the Beneductus trial may be detrimental.

Our study has limitations. Despite adopting strict criteria to restrict its use, 29.8% of babies in the placebo arm received open-label medical therapy, the likely impact of which would have been to increase the PDA closure rate in this group and make it more difficult to demonstrate between-group differences in clinical outcomes. We did not meet our recruitment goal, enrolling 653 participants out of a target of 730. This was partly due to drug non-availability, changes in clinical practice, competing trials and the impact of the COVID-19 pandemic. Although early assessment and randomization was encouraged, the median age at which the first dose of trial medication was administered was 61 hours, a time that was later than in other similar studies.^19 21^ Trial entry up to 72 hours after birth was permitted to allow a pragmatic approach to enrollment and to ensure the availability of an echocardiography assessment. However, it is possible that earlier intervention may have achieved more effective ductal closure.^25 30^

In conclusion, in extreme preterm infants with a large PDA, there was no evidence that early treatment with ibuprofen was associated with an improvement in the composite outcome of death or moderate or severe BPD compared to placebo at 36 weeks post-menstrual age.

Disclosure forms provided by the authors are available with the full text of this article at NEJM.org.

## Supplementary Material

supplement

## Figures and Tables

**Table 1:** Baseline characteristics

Characteristic	Ibuprofen (n = 324)	Placebo (n = 322)
**Maternal characteristics**		
**Mother’s ethnicity— no. (%)**		
White	223 (74.6)	223 (73.6)
Asian	39 (13.0)	45 (14.9)
Black	25 (8.4)	25 (8.3)
		
Other	12 (4.0)	10 (3.3)
Not known	25	19
**Mother’s age— yr**	30.1±6.5	30.2±6.2
**Infant characteristics at randomization**		
Postnatal age (hours)^*^ **—** **IQR**	57.5	56.8
	(43.1, 65.6)	(43.9, 66.7)
< 12 hours— no. (%)	2 (0.6)	2 (0.6)
12 to < 24 hours— no. (%)	15 (4.6)	14 (4.3)
24 to < 48 hours— no. (%)	90 (27.8)	89 (27.6)
48 to < 72 hours— no. (%)	217 (67.0)	217 (67.4)
Gestational age— weeks^*^	26.1±1.5	26.1±1.6
**Mode of birth— no. (%)**		
Vaginal birth – cephalic	141 (43.5)	138 (42.9)
Vaginal birth – breech	50 (15.4)	46 (14.3)
Caesarean section before onset of labour	83 (25.6)	80 (24.8)
Caesarean section after onset of labour	50 (15.4)	58 (18.0)
**Birth weight— grams**	839.9±204.8	852.9±211.3
Sex^*^— no. (%)		
Male	180 (55.6)	175 (54.3)
**APGAR score 5 minutes after birth— no.**	278	288
Median (IQR)	8.0 (6.0, 9.0)	7.0 (6.0, 9.0)
Size of PDA (mm)^*^ — IQR	2.2 (1.9, 2.5)	2.2 (1.9, 2.6)
≥1.5 mm and <2.0 mm— no. (%)	84 (25.9)	82 (25.5)
≥2.0 mm and <3.0 mm— no. (%)	201 (62.0)	201 (62.4)
≥3.0 mm— no. (%)	39 (12.0)	39 (12.1)
Mode of respiratory support^*^— no. (%)		
Invasive ventilation (by endotracheal tube)	206 (63.6)	204 (63.4)
Non-invasive respiratory support only^1^	116 (35.8)	115 (35.7)
Receiving no mechanical ventilation or		
pressure support^2^	2 (0.6)	3 (0.9)
Receiving inotropes*— no. (%)	44 (13.6)	37 (11.5)

Data are presented as mean ±SD or median and interquartile range.

* Denotes factor used in the randomization minimization algorithm

SD denotes standard deviation and IQR interquartile range.

1 Nasal continuous positive airway pressure, nasal ventilation, humidified high flow nasal cannula therapy, or low flow oxygen ≥ 1.1L per minute.

2 In room air, low flow oxygen <1.1L per min, or ambient oxygen.

**Table 2:** Primary and secondary outcomes

Outcome	Ibuprofen (n = 324)	Placebo (n = 322)	Unadjusted effect measure (95% CI)	Adjusted effect measure^1^ (95% CI)
**Primary outcome**				
**Death or moderate or** **severe BPD at 36 weeks’** **postmenstrual age** **^2^** **—** **no./total no. (%)**	220/318 (69.2)	202/318 (63.5)	Risk ratio 1.09 (0.97—1.22)	Risk ratio 1.09 (0.98—1.20) P=0.10
**Secondary outcomes**				
**Death by 36 weeks’** **postmenstrual age** **—** **no./total no. (%)**	44/323 (13.6)	33/321 (10.3)	Risk ratio 1.33 (0.87—2.02)	Risk ratio 1.32 (0.92—1.90)
**Infants survived up to 36** **weeks’ postmenstrual** **age** **— ** **no.**	280	289		
**Moderate or severe BPD** **at 36 weeks’** **postmenstrual age** **—** **no./total no. (%)**	176/274 (64.2)	169/285 (59.3)	Risk ratio 1.08 (0.95—1.23)	Risk ratio 1.09 (0.96—1.23)
**Any intraventricular** **hemorrhage,** **— ** **no. (%)**	137 (42.3)	132 (41.0)		
	
Grade I/II without ventricular dilatation	92 (28.4)	98 (30.4)		
Severe IVH (grade III/IV)^3^	45 (13.9)	34 (10.6)	Risk ratio 1.32 (0.87—2.00)	Risk ratio 1.30 (0.93—1.82)
**Cystic PVL— no. (%)**	15 (4.6)	9 (2.8)	Risk ratio 1.66 (0.74—3.73)	Risk ratio 1.62 (0.69—3.83)
**Treated for retinopathy** **of prematurity** ^4^ **— no.** **(%)**	45 (13.9)	45 (14.0)	Risk ratio 0.99 (0.68—1.46)	Risk ratio 0.98 (0.68—1.42)
**Significant pulmonary** **hemorrhage** ^5^ **— no./total** **no. (%)**	24/322 (7.5)	18/322 (5.6)	Risk ratio 1.33 (0.74—2.41)	Risk ratio 1.39 (0.70 —2.77)
**Treated for acute** **pulmonary hypertension** **with pulmonary** **vasodilator** **— no. (%)**	17 (5.2)	16 (5.0)	Risk ratio 1.05 (0.54—2.05)	Risk ratio 1.04 (0.51—2.13)
**NEC Bell stage II and** **above** ^6^ **— no./total no.** **(%)**	41/323 (12.7)	41/322 (12.7)	Risk ratio 1.00 (0.67—1.49)	Risk ratio 1.01 (0.67—1.51)
**Closed or PDA <1.5mm** **at around 3 weeks of age,** **confirmed by** **echocardiography—** **no./total no. (%)**	176/317 (55.5)	117/316 (37.0)	Risk ratio 1.50 (1.26—1.79)	Risk ratio 1.50 (1.30 —1.74)
**PDA ≥1.5mm at around** **3 weeks of age, not** **treated medically or by** **surgical closure—** **no./total no.** **(%)**	74/321 (23.1)	109/317 (34.4)	Risk ratio 0.67 (0.52—0.86)	Risk ratio 0.67 (0.53—0.85)
**Open-label treatment of** **a symptomatic PDA by** **surgical treatment— no.** **(%)**	9 (2.8)	31 (9.6)	Risk ratio 0.29 (0.14—0.60)	Risk ratio 0.29 (0.18—0.47)
**Discharged home on** **oxygen— no. (%)**	130 (41.3)	123 (39.2)	Risk ratio 1.05 (0.87—1.27)	Risk ratio 1.06 (0.92—1.22)
**Weight gain: change in z** **score between birth and** **discharge—no.**	257	265		
Mean	-1.0±1.0	-1.1±1.0	Mean difference 0.1 (-0.1—0.2)	Mean difference 0.1 (-0.1—0.2)

PVL denotes periventricular leukomalacia, NEC necrotizing enterocolitis, and PDA patent ductus arteriosus.

The Bell classification system for necrotizing enterocolitis ranges from stage I to stage III with a higher stage indicating more severe illness.

1 Adjusted for minimization factors (size of the PDA, gestational age at birth, age at randomization, sex, center, multiple births, mode of respiratory support at randomization, and receiving inotropes or not at the time of randomization) and the correlation between siblings from multiple births, where technically possible. Center was treated as a random effect in the models, and all other factors as fixed effects.

2 In the placebo group, 1 infant was withdrawn before 36 weeks and 3 did not have oxygen requirement information available around 36 weeks. In the ibuprofen group, 1 infant was lost to follow-up before 36 weeks and 5 did not have oxygen requirement information available around 36 weeks.

3 With ventricular dilatation or intraparenchymal abnormality.

4 In at least one eye.

5 Fresh blood in endotracheal tube with increase in respiratory support.

6 Confirmed by radiography and/or histopathology.

**Table 3:** Process outcomes

Outcome	Ibuprofen (n = 324)	Placebo (n = 322)
**Did not receive allocated intervention— no.** **(%)**	7 (2.2)	11 (3.4)
**Incomplete trial medications— no. (%)**	41 (12.7)	34 (10.6)
**Doses received— no. (%)**		
0	7 (2.2)	9 (2.8)
1	17 (5.2)	11 (3.4)
2	17 (5.2)	14 (4.3)
3	283 (87.3)	288 (89.4)
**Reason treatment stopped early^1^— no./total** **no. (%)**		
Clinical decision	38/41 (92.7)	26/34 (76.5)
Parental request	0	1/34 (2.9)
Baby died	2/41 (4.9)	2/34 (5.9)
Missed dose(s) in error	1/41 (2.4)	2/34 (5.9)
Outside of trial period	0	2/34 (5.9)
Transferred out of recruiting site	0	1/34 (2.9)
**Age at first dose (hours) — IQR** ^2^	61 (47 to 68)	61 (48 to 69)
0 to <24 hours— no./total no. (%)	14/316 (4.4)	14/313 (4.5)
24 to <48 hours— no./total no. (%)	68/316 (21.5)	65/313 (20.8)
48 to <72 hours— no./total no. (%)	220/316 (69.6)	225/313 (71.9)
≥ 72 hours— no./total no. (%)	14/316 (4.4)	9/313 (2.9)
**Received 2**^nd^** or 3**^rd^** dose outside of dosing** **window— no./total no. (%)**	5/297 (1.7)	4/301 (1.3)
**ECHO not done around 3 weeks of age** ^3^ **— no./total no. (%)**	65 (22.3)	60 (19.9)
**Oxygen reduction test not done when baby** **was eligible**^4^**— no./total no. (%)**	13/86 (15.1)	13/94 (13.8)

1 Fewer than 3 doses.

2 Of those who received at least one dose.

3 Within 18 to 24 days of birth, excluding deaths that occurred before the 3-week echocardiogram could be performed: 33 patients assigned to ibuprofen and 21 assigned to placebo.

4 As a proportion of babies eligible for the test.
