# Supplementary material for: Trial of Selective Early Treatment of Patent Ductus Arteriosus with Ibuprofen
Source: N Engl J Med. Author manuscript; Available in PMC 2024 Mar 29. (PMC7615774; doi:10.1056/NEJMoa2305582)
Supplement: supplement [file EMS194589-supplement-supplement.pdf]

Supplementary Appendix

Supplement to: Gupta S, Subhedar N, Bell JL et al. Trial of Selective Early Treatment of Patent Ductus Arteriosus with Ibuprofen. New Engl J Med 2024. DOI: XXX

This appendix has been provided by the authors to give readers additional information about their work.

Table of Contents

Baby-OSCAR Collaborative Group 3

National Perinatal Epidemiology Unit Clinical Trials Unit, University of Oxford, UK 4

Institute of Applied Health Research, University of Birmingham, Birmingham, UK 4

Research & Development Department, University Hospital of North Tees, Stockton, UK 4

Study Committees 5

Independent Trial Steering Committee Members 5

Independent Data Monitoring Committee Members 5

Acknowledgements 5

Methods 5

Details of sample size and power calculation 6

Details of primary outcome model 6

Figures 7

Figure S1: Oxygen reduction test flow chart 7

Figure S2: Model diagnostics for the fitted robust Poisson regression model for the primary outcome 8

Figure S3: Forest plot of moderate/severe BPD at 36 weeks' subgroup analysis 10

Figure S4: Forest plot of death by 36 weeks' subgroup analysis 11

Figure S5: Time from randomization to open-label treatment 11

Tables 12

Table S1: Inclusion and Exclusion Criteria 12

Table S2: Trial intervention 12

Table S3: Criteria for open-label medical or surgical treatment after enrollment 13

Table S4: Severity-Based Criteria for BPD 13

Table S5: Primary and Secondary Outcomes 14

Table S6: Trial Assessments 17

Table S7: Independent variables included in the primary outcome model 18

Table S8: Additional baseline characteristics 19

Table S9: Secondary analysis: Restricted analysis excluding infants who received open label medical treatment[1] without meeting the specified criteria 21

Table S10: Additional process outcomes 21

Table S11: PDA ≥ 1.5mm at around 3 weeks by gestational age 22

Table S12: Post-hoc exploratory analysis: Timing of open-label treatment from randomisation 22

Table S13: Additional secondary outcomes 23

Table S14: Safety Events (between the first dose of trial medication and 7 days after the last dose) 25

CONFIDENTIAL

Table S15: Unforeseeable serious adverse events 26

Table S16: Representativeness of Study Participants 28

References 29

CONFIDENTIAL

Baby-OSCAR Collaborative Group

The following investigators, research nurses, and hospitals participated in the Baby-OSCAR Study. Sites are listed alphabetically.

Recruiting site for Baby-OSCAR	PI(s)	Research Staff
Aberdeen Maternity Hospital	Dr Saulius Satas	Margaret Connon, Stephen Main, Susan MacFarlane
Addenbrookes's Hospital, Cambridge	Dr Anthony Wilfred Ross Kelsall	Katherine Bradly Russell, Helen Shelley, Beth Berthlecon, Dr Sajeev Job
Arrowe Park Hospital, Wirral	Dr Anand Kamalanathan,	Sharon Hughes, Lucy Lewis, Dr Aung Soe
Birmingham Heartlands Hospital	Dr Jaideep Singh	Eve Irvine, Katie Price, Laura Thrasyvoulou, Juneka Begum, Jacqueline Daglish
Birmingham Women's Hospital	Dr Vishna Rasiah<br>Dr Anju Singh	Rachel Jackson, Efygenia Kotsia, Amy Woodhead, Abby Twiss, Maxine Heather Barrow, Elizabeth Simcox
Bradford Royal Infirmary	Dr Sam Wallis	Rachel Wane
Burnley General Hospital	Dr Savithiri Sivashankar	Emily Andrews, Heather Collier, Dr Chi-Ning Gerrard, Caroline Cowman, Bev Hammond, Frances Pickering
Derriford Hospital ,Plymouth	Dr Robert John Madar,	Sarah-Jane Sharman, Alison Stolton
James Cook University Hospital, Middlesbrough	Dr Jonathan Wyllie	Caroline Buckley, Ms Amanda Forster, Helena Smith, Suzanne Bell,
Jessop Wing Hospital, Sheffield	Dr Porus Bustani	Pauline Bayliss, Rachel Sellars, Lynne Smart, Liz Taylor, Pauline Bayliss, Beth Lally
Leeds General Infirmary	Dr Lawrence Miall	Nicola Balatoni, Suzanne Laing, Collette Spencer, Sarah Thornton, Lindsay Uryn, Dr Laura Dalton, Dr Katherine Pettinger, Charlotte Reilly
Leicester Royal Infirmary	Dr Jonathan Cusack	Marie Hubbard, Rosalind Astles, Maria Sharpe, Jennifer Smith
Liverpool Women's Hospital	Dr Nimish Subhedar	Karen Harvey, Joanne Windrow, Patrick McGowan, Amy Beasley
Luton & Dunstable University Hospital	Dr Sateeshkumar Somisetty	Yvonne Millar, Olaitan Adesiyan, Jenny Baker
Medway Maritime Hospital	Dr Santosh Pattnayak	Helen Harizaj, Ms Aimee Harris, Sarah Jones, Alison Youdale, Dr Aung Soe
Norfolk and Norwich University Hospital	Dr Rahul Roy	Samantha Claire, Dr Supriya Bhoomaiah, Karen Few, Katherine Lloyd, Amy Nichols, Laura Playne
Queen Charlotte's and Chelsea Hospital	Dr Jay Banerjee	Batia Gourin, Zoe McClure, Kirupalini Mariampillai, Dr Sundar Satyamurthy
Royal Infirmary of Edinburgh	Dr Christopher Kissack	Sally Yip, Lynn Clark

CONFIDENTIAL

Recruiting site for Baby-OSCAR	PI(s)	Research Staff
Royal Jubilee Maternity Hospital, Belfast	Dr Bharathi Rao	Eileen Killen, Jennifer McGowan, Muriel Millar, Mary O'Neill, Angela Abbate, Rachel Anderson, Julie Brown, Patrick Lawlor, Judith Ratcliffe, Eileen Rogers
Royal Preston Hospital, Preston	Dr Akaolisa Egbeama	Joanna Lees, Claire Lodge, Natalie Morgan, Dr Raju Narasimhan, Paula Sugden
Royal Victoria Infirmary Newcastle	Dr Sundeep Harigopal	Julie Groombridge, Tracey Downes
St George's Hospital, London	Dr Donovan Duffy	Naomi Hayward, Dr Anay Kulkarni
St Mary's Hospital, London	Dr Jay Banerjee	Batia Gourin, Zoe McClure, Izabela Andrzejewska, Kirupalini Mariampillai, Vania Oliveira, Dr Sundar Sathiyamurthy
St Mary's Hospital, Manchester	Dr Arindam Mukherjee	Nicola Booth, Karen Dockery, Clare Jennings, Louise Weaver-Lowe, Katherine Birchall
Sunderland Royal Hospital,	Dr Majid Abu-Harb	Natalie Talbot, Paul Corrigan
The Grange University Hospital	Dr Siddhartha Sen	Alison Davies, Angela Harris
The Royal London Hospital	Dr Ajay Sinha	MaySze Chang, Caroline Francia, Ivone Lancoma-Malcolm, Gail Falder, Dr Rainer Ebel
University Hospital Coventry	Dr Mrinalini Rajimwale	Francesca Brewer, Rebecca Grenfell, Nicola Watts, Laura Wild, Nicolas Aldridge, Susan Dale, Jo Gmerek, Kerri McGowan.
University Hospital of North Tees	Dr Samir Gupta<br>Dr Sundaram Janakiraman	Alex Ramshaw, Wendy Cheadle, Dr Harikumar
Watford General Hospital	Dr Nazakat Merchant	Shabana Malik, Suminthra Naidu, Rona Verdadero
William Harvey Hospital, Ashford	Dr Amit Gupta	Shermi George, Claire Moloney, Vimal Vasu.
Wishaw General Hospital	Dr Gopala Krishnan	Denise Vigni

National Perinatal Epidemiology Unit Clinical Trials Unit, University of Oxford, UK
Jennifer Bell, Ursula Bowler, Christina Cole, Kerrianne Dempster, Clare Edwards, Pollyanna Hardy, Nina Jamieson, Edmund Juszczak, Ann Kennedy, Andy King, Marketa Laube, Louise Linsell, David Murray, Heather O'Connor, Charles Roehr, Kayleigh Stanbury, Julia Sutton, Joy Wiles.

Institute of Applied Health Research, University of Birmingham, Birmingham, UK
Tracey Roberts, Chidubem Okeke Ogwulu

Research & Development Department, University Hospital of North Tees, Stockton, UK
Jane Greenaway, Pauline Shephard, Dr Volker Straub, Dr Justin Carter

CONFIDENTIAL

Study Committees

Independent Trial Steering Committee Members

Ben Snook
Dr Denis Azzopardi
Emeritus Professor Michael Weindling (Chair)
Dr Narender Aladangady
Sophie Welch
Dr Tim Clayton

Independent Data Monitoring Committee Members

Professor Alan Montgomery
Professor David Edwards (Chair)
Dr Heike Rabe

Acknowledgements

We thank all the families, infants, and hospital staff at recruiting and continuing care sites who participated in the trial. We acknowledge independent advice from Dr Nicholas Evans and Dr Steven M. Donn in planning the trial.

Methods

The trial was conducted in accordance with the principles of the Good Clinical Practice guidelines of the International Conference on Harmonisation and received approval from the United Kingdom Medicines and Healthcare Products Regulatory Agency, the NHS Health Research Authority, and East Midlands–Nottingham 2 Research Ethics Committee.

The initial version of the manuscript was drafted by the first and last authors, developed by the writing committee and approved by all members of the Trial Steering Committee and the Data Monitoring Committee. The funders had no role in the analysis of the data, the preparation or approval of the manuscript, or the decision to submit the manuscript for publication. The members of the writing committee vouch for the completeness and accuracy of the data and for the fidelity of the trial to the protocol and statistical analysis plan.

Supplementary Statistical Methods

The enrolling center was treated as a random effect in the models, and all other factors as fixed effects. Since models only adjusted for minimization factors required at randomization, covariates in the regression analyses had no missing data. Both crude and adjusted effect estimates are presented, with primary inference based on the adjusted estimate. Demographic and clinical data are presented as counts and percentages for categorical variables, means and standard deviations for normally distributed continuous variables and medians and interquartile ranges for other continuous variables.

CONFIDENTIAL

A secondary analysis on the primary outcome and its components excluded infants who received open-label medical treatment up to 36 weeks or discharge (if sooner) without meeting pre-specified criteria. A pre-specified subgroup analysis on necrotizing enterocolitis was conducted by the size of the PDA.

Details of sample size and power calculation

Evidence from the TIPP trial suggests that the risk of death or bronchopulmonary dysplasia (BPD) in extremely low birth weight babies at 36 weeks postmenstrual age allocated placebo is 52% (95% CI, 48 to 56)[1 2]. However, this trial investigated the effect of prophylactic treatment and included all babies weighing 500 – 999 g. More recent information using data derived from the latest report of Neonatal Survey Database from the Trent region[3] provides an approximate rate of death by or BPD at 36 weeks postmenstrual age of 53% for all babies admitted to the neonatal unit. These babies would have been treated according to clinical judgement and therefore a proportion of them would have been treated with ibuprofen. Given that the risk of death or BPD in babies with an echocardiographically confirmed large patent ductus arteriosus (PDA) is inherently higher, it is estimated that the risk in this group is 60%.

Su et al. (2008)[4] compared ibuprofen to indomethacin in babies ≤28 weeks' gestation having a PDA who were less than 24 hours old. The combined outcome of death within 30 days or BPD at 36 weeks postmenstrual age was observed to be 42% (95% CI, 29 to 55).

It is therefore expected, given that babies will be enrolled up to 72 h after birth, that the treatment group incidence of death or BPD at 36 weeks postmenstrual age will be approximately 48% in the intervention arm. This would imply an absolute risk reduction of 12% (60 to 48) in the primary outcome of the trial for babies randomised to treatment compared to placebo, which is considered a clinically important difference.

Details of primary outcome model

Mixed effects Poisson regression model with a robust variance estimator was used, as a log binomial regression model failed to converge. The mode was adjusted for minimization factors at randomisation (PDA size; gestational age at birth; age; sex; multiple birth; mode of respiratory support; receiving inotropes) as fixed effects, and trial site and multiple cluster (i.e. an identifier used to link siblings from multiple births) as random effects.
The mathematical form of the model for the primary outcome is:

$$\log(\mu \quad) = {}_{0} + {}_{1}x_{\ 1} + {}_{2}x_{\ 2} + {}_{3}x_{\ 3} + {}_{4}x_{\ 4} + {}_{5}x_{\ 5} + {}_{6}x_{\ 6} + {}_{7}x_{\ 7} + {}_{8}x_{\ 8} + u_{\ i} +$$

for site *k*, multiple cluster *j*, and patient *i*.

The variables included in the model are described in Table S7.

CONFIDENTIAL

Figures

Figure S1: Oxygen reduction test flow chart

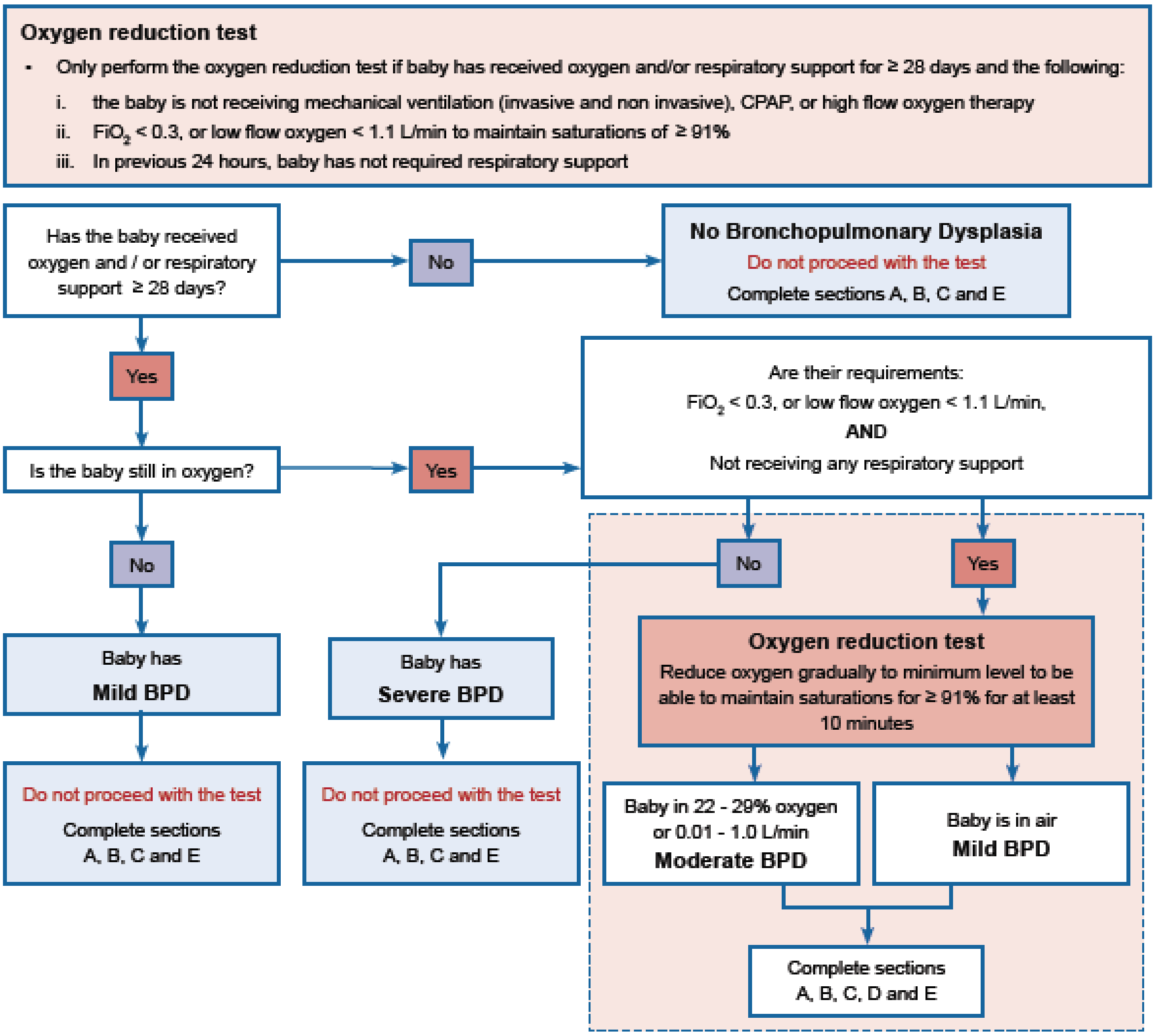

FiO_2 denotes the fraction of inspired oxygen and CPAP continuous positive airway pressure.

Figure S2: Model diagnostics for the fitted robust Poisson regression model for the primary outcome

The following model diagnostics were assessed for the robust Poisson regression model for the primary outcome:

- A plot of deviance residuals for each allocation to assess the distribution of residuals (Figure S2a), respecting the response distribution assumption for the Poisson model.
- A plot of deviance residuals vs fitted values to assess linearity between the transformed expectation of Y and the predictors $x_1, \ldots, x_7$ (Figure S2b). No trend was seen respecting the linearity assumption in the Poisson regression.
- Normality of deviance residuals was assessed and the pattern, respecting the response distribution assumption for the Poisson model (Figure S2c).
- In general, individual plots of the deviance residuals versus independent variables in the model suggested no trend, as illustrated using the Locally Weighted Scatterplot Smoothing (LOWESS) curves (Figure S2d).

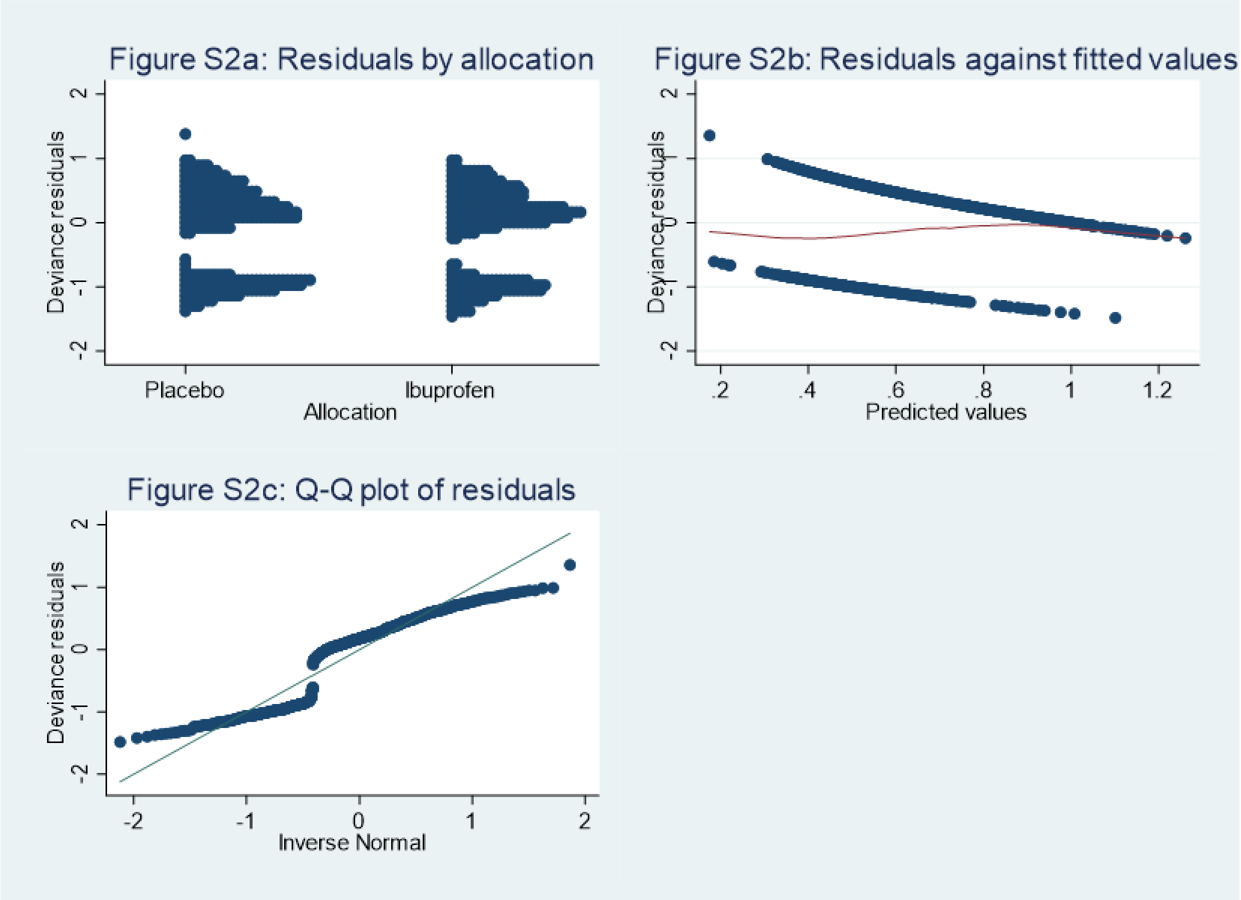

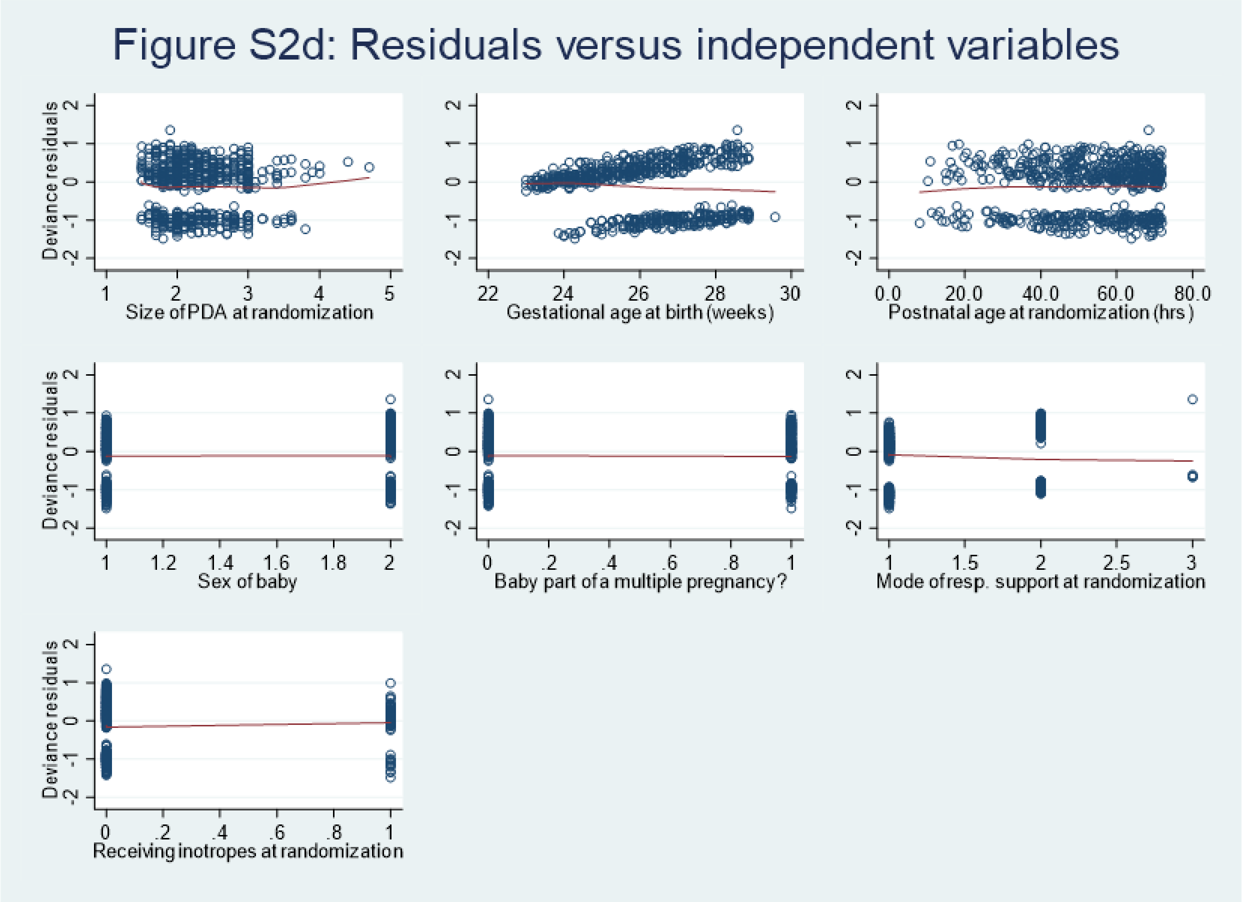

CONFIDENTIAL

Figure S3: Forest plot of moderate/severe BPD at 36 weeks' subgroup analysis

Note: The mode of respiratory support has been collapsed into fewer subgroups than pre-specified due to the low number of patients in some subgroups.

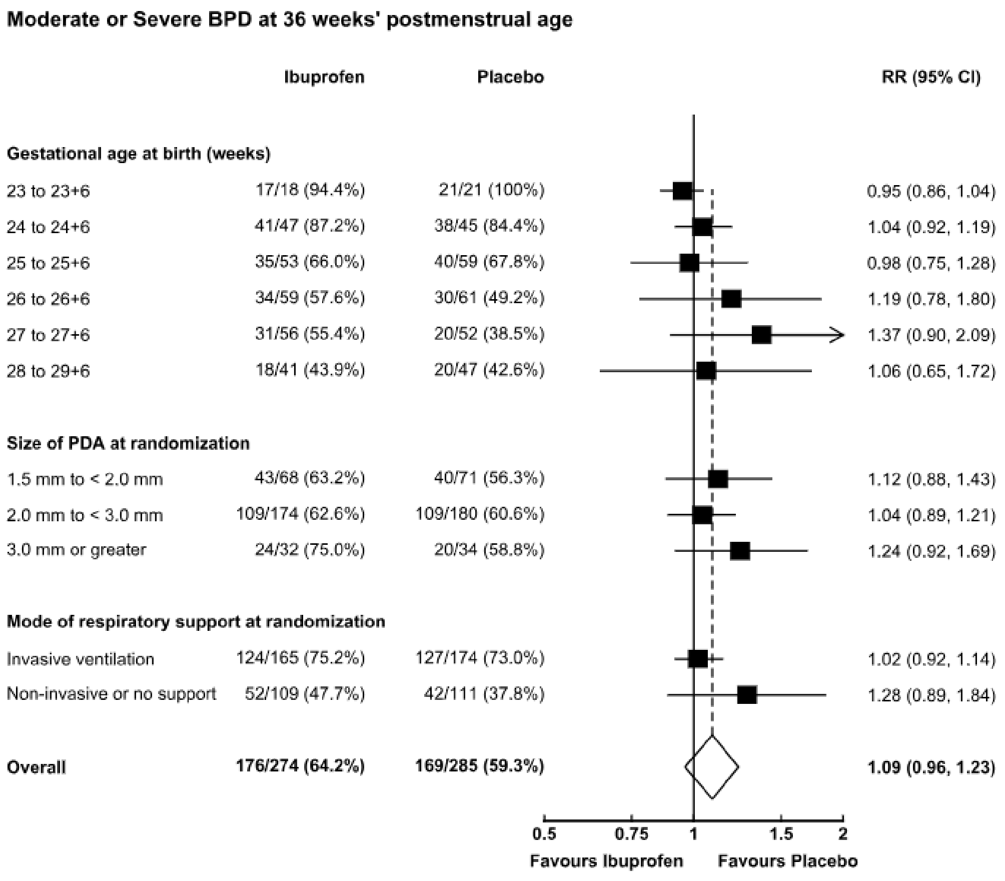

Risk ratios (RR) and 95% confidence intervals (CI) were obtained from an interaction term between treatment assignment and the subgroup characteristic of interest, in a log binomial model adjusted for the size of the PDA at randomization, gestational age at birth, age at randomization, sex, multiple birth, mode of respiratory support at randomization, receiving inotropes at time of randomization, and center as a random effect, and clustered by siblings to account for correlation between multiple births.
No adjustments for multiplicity of testing have been applied and therefore interpretation of the confidence intervals should not be used to assess treatment effect.

Figure S4: Forest plot of death by 36 weeks' subgroup analysis

Note: Subgroups have been collapsed into fewer groups than pre-specified due to the low numbers of patients in some groups.

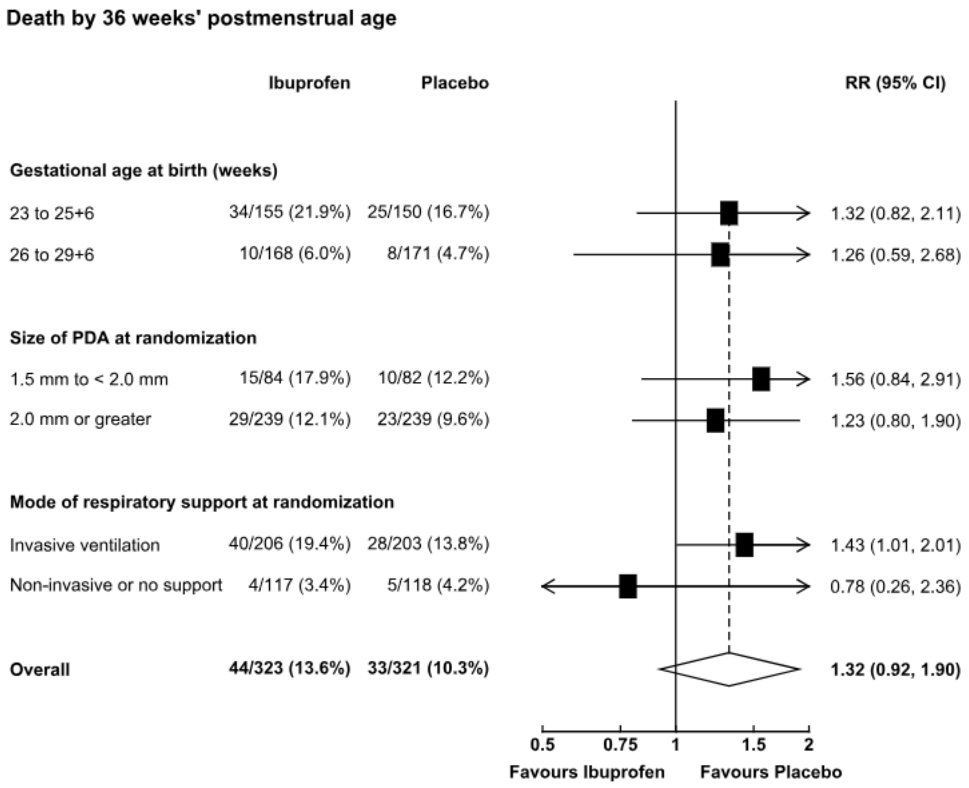

RR denotes risk ration and CI confidence intervals. Risk ratios and 95% CI were obtained from an interaction term between treatment assignment and subgroup characteristic of interest, in a log binomial model adjusted for size of PDA at randomization, gestational age at birth, age at randomization, sex, multiple birth, mode of respiratory support at randomization, receiving inotropes at time of randomization, and center as a random effect, and clustered by siblings to account for correlation between multiple births. No adjustments for multiplicity of testing have been applied and therefore interpretation of the confidence intervals should not be used to assess treatment effect.

Figure S5: Time from randomization to open-label treatment

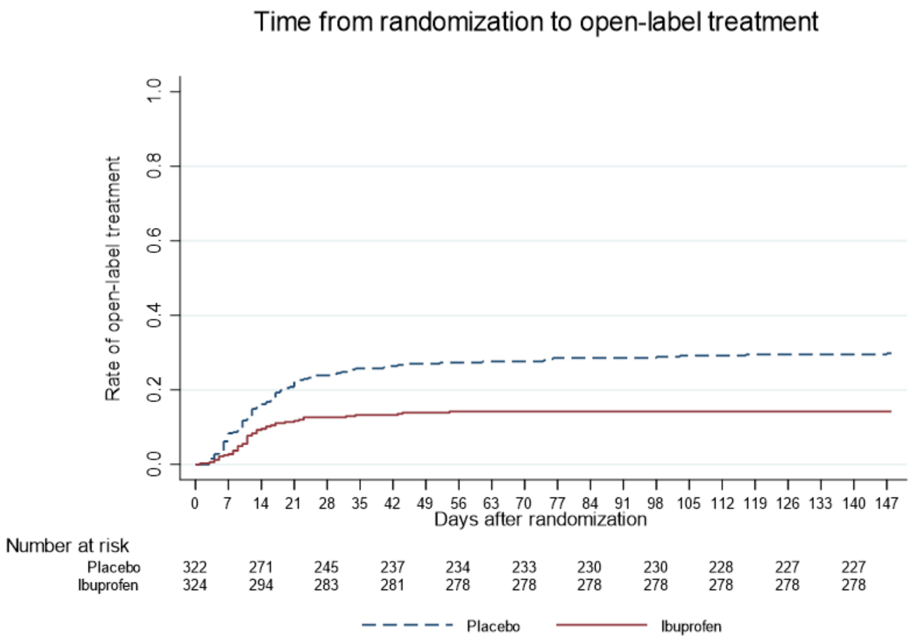

Tables

Table S1: Inclusion and Exclusion Criteria

**Inclusion Criteria**	**Exclusion Criteria**
Babies will be considered eligible for inclusion into the trial if they are:<br>• Born at 23^{+0}–28^{+6} weeks' gestation<br>• Less than 72 h old<br>• Confirmed by echocardiography to have a large PDA which is at least 1.5 mm in diameter (determined by gain optimised colour Doppler),<br>*And*<br>• has unrestrictive pulsatile (left to right) flow (ratio of flow velocity in PDA Maximum (V_{max}) to Minimum (V_{min}) > 2:1)) or, growing flow pattern (< 30% right to left), and no clinical concerns of pulmonary hypertension<br>In addition:<br>• The responsible clinician is uncertain about whether the baby might benefit from treatment to close the PDA<br>• Written informed consent is obtained from the parent(s)	Babies will be excluded from participation in the trial if they have:<br>• No realistic prospect of survival<br>• Severe congenital anomaly<br>• Clinical or echocardiography suspicion of congenital structural heart disease that contraindicates treatment with ibuprofen<br>• Other conditions that would contraindicate the use of ibuprofen (active bleeding especially intracranial or gastrointestinal bleeding, coagulopathy, thrombocytopenia (platelet count < 50,000), renal failure, life threatening infection, pulmonary hypertension, known or suspected necrotising enterocolitis (NEC))<br>• Indomethacin, ibuprofen, or paracetamol administration after birth

Table S2: Trial intervention

**Timing of intervention**	**Interventional medicinal product – Ibuprofen (5mg/ml solution)**	**Placebo - 0.9% Sodium Chloride**
Ist dose	10 mg/kg (2 ml/kg)	2 ml/kg
2nd dose<br>(after 24 hours of 1st dose)	5 mg/kg (1 ml/kg)	1 ml/kg
3rd dose<br>(after 24 hours of 2nd dose)	5 mg/kg (1 ml/kg)	1 ml/kg

Doses were calculated based on birth weight and administered as a short infusion over 15 minutes, diluted to the appropriate volume with dextrose or saline. The first dose is administered soon after randomization and within 72 hours of birth.

CONFIDENTIAL

Table S3: Criteria for open-label medical or surgical treatment after enrollment

Clinical Criteria	Echocardiography Criteria
Inability to wean on ventilator (ventilated for at least 7 days continuously) and any of the following:<br>Inability to wean oxygen; or<br>Persistent hypotension; or<br>Pulmonary hemorrhage; or<br>Signs of cardiac failure	Presence of a large PDA with a ductal dimension of ≥ 2.0 mm<br>AND<br>Unrestrictive pulsatile left to right flow in PDA<br>AND<br>Presence of a hyperdynamic circulation OR ductal steal

Table S4: Severity-Based Criteria for BPD

Severity-Based Diagnostic Criteria for BPD

Time point of assessment:	36 weeks of postmenstrual age
Therapy with oxygen > 21% and/or respiratory support for ≥ 28 days and the following:	
Mild BPD;	Baby is breathing room air
Moderate BPD;	Baby is in 22–29% oxygen, or 0.01–1.0 L/min
Severe BPD;	FiO_2 ≥ 0.3, or low flow oxygen ≥ 1.1 L/min, or the baby is receiving any respiratory support (ventilation, CPAP, or high flow oxygen therapy) to achieve oxygen saturation ≥ 91%

FiO_2 denotes the fraction of inspired oxygen and CPAP continuous positive airway pressure.

The need for oxygen is subjective and hence oxygen dependency will be confirmed using an 'oxygen reduction test'. This is based on the threshold at which the baby is able to maintain oxygen saturations ≥ 91% whilst breathing in air or at a given minimum FiO_2. Babies unable to achieve this will be considered to be oxygen dependent. This test will only apply to those babies whose oxygen requirements are < 0.3, or low flow oxygen < 1.1 L/min, and who have not received any additional respiratory support in the previous 24 hours. Babies outside of this will not be tested, but their oxygen requirements will be collected.

CONFIDENTIAL

Table S5: Primary and Secondary Outcomes

Due to the multiple number of short-term outcomes and the absence of correction for multiple testing, statistical inference is restricted to a predefined list of tested outcomes. Summary data by trial arm is provided for all other outcomes, but statistical tests or the calculation of confidence intervals are not performed. Secondary outcomes are divided into short- and long-term outcomes. Note that outcomes listed appear in a different order than as described in the Study Protocol.

Tested outcomes
**Primary outcome**<br><br>A composite of death by 36 weeks of postmenstrual age, or moderate or severe BPD at 36 weeks of postmenstrual age (see Table S4).
**Secondary outcomes**<br><br>**Short term outcomes:**<br>• Death by 36 weeks of postmenstrual age<br>• Moderate or severe BPD at 36 weeks of postmenstrual age (see Table S4)<br><br>Incidence or duration of the following up to discharge:<br>• Severe intraventricular hemorrhage (IVH) (grade III/IV with ventricular dilatation or intraparenchymal abnormality)<br>• Cystic periventricular leukomalacia (PVL)<br>• Babies treated for retinopathy of prematurity (ROP)<br>• Significant pulmonary hemorrhage (fresh blood in endotracheal tube with increase in respiratory support)<br>• Treated for pulmonary hypertension with pulmonary vasodilator<br>• NEC definitive and/or complicated (Bell stage II and above) confirmed by radiology and/or histopathology<br>• Closed or non-significant PDA (<1.5 mm) at around 3 weeks of age (range of 18 – 24 days), confirmed by ECHO<br>• PDA ≥ 1.5 mm at around 3 weeks' (range of 18 – 24 days)<br>• Open-label treatment of a symptomatic PDA by surgical treatment<br>• Discharge home on oxygen<br>• Weight gain: a change in z score between birth and discharge (or death if sooner)<br><br>**Long Term Outcomes assessed at 24 months of age corrected for prematurity*:**<br>• Survival without moderate or severe neurodevelopmental impairment (main long-term outcome)

CONFIDENTIAL

- Death
- Moderate or severe neurodevelopmental impairment in survivors
- Survival without respiratory morbidity (defined as any 2 or more of need for oxygen or respiratory support; presence of a persistent cough and/ or wheeze; need for regular treatment for respiratory illness; 4 or more unscheduled attendances at hospital/ GP for respiratory problems; 1 or more readmission to hospital for respiratory problems)
- Duration of oxygen supplementation from randomization

Untested outcomes (described only)

Secondary outcomes

Short term outcomes:

- Severity of BPD at 36 weeks of postmenstrual age (see Table S4)

Incidence or duration of the following up to discharge:

- Non-cystic PVL
- Hydrocephalus
- NEC requiring surgery
- Gastrointestinal bleeding (leading to investigation or clinical treatment) within 7 days of the first dose of trial drug administration
- Spontaneous intestinal perforation
- Medical open-label treatment of a symptomatic PDA with a COX inhibitor
- Administration and duration of inotropic support
- Total duration of respiratory support
- Invasive ventilation through an endotracheal tube
- Non-invasive support through, nasal CPAP, nasal ventilation, humidified high flow nasal cannula therapy, or low flow oxygen ≥ 1.1L/min
- Duration of initial hospitalization (birth to discharge home)
- Postnatal steroid use for chronic lung disease
- Tolerance of ibuprofen treatment within the foreseeable SAE reporting range, described in the protocol, section 9.1.4
- Head circumference: a change in head size z score between randomization and discharge (or death if sooner)

Long Term Outcomes assessed at 24 months of age corrected for prematurity*:

- Individual components of respiratory morbidity, in survivors, as follows:
  - Need for oxygen or respiratory support
  - Presence of a persistent cough and/ or wheeze

CONFIDENTIAL

• Need for regular treatment for respiratory illness<br>• 4 or more unscheduled attendances at hospital/ GP for respiratory problems<br>• 1 or more readmissions to hospital for respiratory problems
A cost-effectiveness analysis will be conducted of deaths and BPD events avoided and national health services used up to 2 years of age corrected for prematurity*

*To be reported in separate manuscript

CONFIDENTIAL

Table S6: Trial Assessments

Procedure	Baby Hospitalization					
	Screening[1]	Trial Entry and Treatment (days 1–3)	Up to 7 days after trial medication	3 weeks of Age	36 weeks of PMA[2]	Discharge
**Demography[3]**		✓				✓
**Echocardiogram/Colour Doppler[4]**	✓			✓		
**Confirmation of Eligibility**	✓					
**Consent**		✓				
**Randomization[5]**		✓				
**Ibuprofen or Placebo Dosing[6]**		✓				
**IVH or PVL ultrasound scans[2]**			✓		✓	
**NEC**						✓
**Oxygen Reduction Test**					✓	
**SAEs[7]**		✓	✓			
**Concomitant Medication[8]**	✓	✓		✓	✓	✓

SAE denotes serious adverse event.

[1] Screening assessments to be completed sufficiently in advance to enable randomization and dosing within 72 hours of birth. If consent cannot be obtained before echocardiographic evaluation for eligibility, echocardiographic assessment should continue and consent obtained when possible if a baby is deemed eligible.

[2] If a baby transfers from the recruiting site to a continuing care site for on-going care details of any scan were considered helpful.

[3] Demography and medications will be assessed through the Parent Report of Children's Abilities-Revised (PARCA-R) and other questionnaires.

[4] An echocardiogram scan will be performed when the baby reaches around 3 weeks of age (range of 18 – 24 days) or at hospital discharge if discharged earlier.

[5] Randomization to be completed sufficiently in advance to enable dosing within 72 hours of birth.

[6] Initial trial drug administrations to be given soon after randomization, after 6 hours of age and within 72 hours of birth. Subsequent doses to be administered 24 hours after the initial dose.

[7] Only adverse events which are serious will be recorded from first dose until 7 days after trial medication. Only unforeseeable SAEs will be reported.

[8] Concomitant medications to be recorded only in relation to unforeseeable SAEs. In the event of an unforeseeable SAE all concomitant medication, including medication given to the baby’s mother, 7 days prior to the onset of the event to the time of its resolution must be recorded on the SAE form.

CONFIDENTIAL

Table S7: Independent variables included in the primary outcome model

Variable	Effect type	Functional form
Randomized allocation	Fixed effect	Binary:<br>Placebo: x_1 = 0<br>Ibuprofen: x_1 = 1
Size of PDA at randomization	Fixed effect	Categorical:<br>1.5 mm to < 2.0 mm: x_2 = 0<br>2.0 mm to < 3.0 mm: x_2 = 1<br>≥ 3.0 mm: x_2 = 2
Gestational age at birth	Fixed effect	Categorical:<br>23 to 23^{+6} weeks: x_3 = 0<br>24 to 24^{+6} weeks: x_3 = 1<br>25 to 25^{+6} weeks: x_3 = 2<br>26 to 26^{+6} weeks: x_3 = 3<br>27 to 27^{+6} weeks: x_3 = 4<br>28 to 28^{+6} weeks: x_3 = 5
Age at randomization	Fixed effect	Categorical:<br>< 12 hours: x4 = 0<br>12 to < 24 hours: x_4 = 1<br>24 to < 48 hours: x_4 = 2<br>48 to < 72 hours: x_4 = 3
Sex	Fixed effect	Binary:<br>Female: x_5 = 0<br>Male: x_5 = 1
Multiple birth	Fixed effect	Binary:<br>Singleton: x_6 = 0<br>Multiple: x_6 = 1
Mode of respiratory support at randomization	Fixed effect	Categorical:<br>Invasive ventilation: x_7 = 0<br>Non-invasive respiratory support: x_7 = 1<br>Receiving no mechanical ventilation, or pressure support: x_7 = 2
Receiving inotropes at randomization	Fixed effect	Binary:<br>No: x_8 = 0<br>Yes: x_8 = 1
Trial site	Random intercept	Categorical: one level per site k, u_k
Multiple cluster ID	Random intercept	Categorical: Infants each assigned an identifier according to their family j, i.e. the ID is equal for siblings, within each site, v_{jk}

CONFIDENTIAL

Table S8: Additional baseline characteristics

Characteristic	Ibuprofen (n = 324)	Placebo (n = 322)
**Maternal characteristics**		
**Deprivation index[1] — no.**	271	282
1 (Least deprived) — no. (%)	103 (38.0)	101 (35.8)
2 — no. (%)	57 (21.0)	75 (26.6)
3 — no. (%)	52 (19.2)	40 (14.2)
4 — no. (%)	37 (13.7)	41 (14.5)
5 (Most deprived) — no. (%)	22 (8.1)	25 (8.9)
Missing or not defined — no.	53	40
**Antenatal steroid use**		
Any — no. (%)	293 (90.7)	290 (90.9)
< 24 hours before birth[2] — no. (%)	101 (34.5)	102 (35.2)
≥ 24 hours before birth — no. (%)	192 (65.5)	188 (64.8)
Missing — no.	1	3
**Antenatal COX inhibitor use — no. (%)**	43 (13.5)	36 (11.4)
Missing — no.	6	5
**Antenatal magnesium sulfate use for neuroprotection — no. (%)**	236 (76.1)	245 (79.3)
Missing — no.	14	13
**Infant characteristics at randomization**		
**Born in enrolling center — no. (%)**	273 (84.3)	277 (86.0)
**Enrolling center* — no. (%)**		
1	30 (9.3)	29 (9.0)
2	28 (8.6)	24 (7.5)
3	22 (6.8)	20 (6.2)
4	19 (5.9)	19 (5.9)
5	17 (5.2)	16 (5.0)
6	19 (5.9)	14 (4.3)
7	15 (4.6)	18 (5.6)
8	13 (4.0)	19 (5.9)
9	14 (4.3)	17 (5.3)
10	14 (4.3)	10 (3.1)
11	11 (3.4)	11 (3.4)
12	12 (3.7)	9 (2.8)
13	11 (3.4)	9 (2.8)
14	8 (2.5)	10 (3.1)
15	7 (2.2)	10 (3.1)
16	5 (1.5)	11 (3.4)
17	10 (3.1)	6 (1.9)
18	8 (2.5)	8 (2.5)
19	6 (1.9)	9 (2.8)
20	8 (2.5)	6 (1.9)
21	5 (1.5)	8 (2.5)
22	9 (2.8)	3 (0.9)
23	4 (1.2)	8 (2.5)
24	5 (1.5)	7 (2.2)
25	5 (1.5)	6 (1.9)
26	6 (1.9)	4 (1.2)
27	4 (1.2)	4 (1.2)
28	3 (0.9)	2 (0.6)

CONFIDENTIAL

Characteristic	Ibuprofen (n = 324)	Placebo (n = 322)
29	3 (0.9)	2 (0.6)
30	2 (0.6)	2 (0.6)
31	1 (0.3)	1 (0.3)
**Gestational age (weeks)* — no. (%)**		
23 to < 24 weeks	30 (9.3)	29 (9.0)
24 to < 25 weeks	59 (18.2)	58 (18.0)
25 to < 26 weeks	66 (20.4)	63 (19.6)
26 to < 27 weeks	66 (20.4)	68 (21.1)
27 to < 28 weeks	61 (18.8)	56 (17.4)
28 to < 29 weeks	42 (13.0)	47 (14.6)
≥ 29 weeks	0	1 (0.3)
**Forceps or Ventouse used in delivery — no. (%)**	4 (1.2)	2 (0.6)
Missing	0	2
**Main cause of preterm birth — no. (%)**		
Preterm pre-labor rupture of membranes (PPROM)	112 (34.6)	101 (31.4)
Preterm labor (without PROM)	116 (35.8)	118 (36.6)
Antepartum hemorrhage[3]	34 (10.5)	39 (12.1)
Hypertension[4]	6 (1.9)	5 (1.6)
Pre-eclampsia	8 (2.5)	15 (4.7)
Sepsis	7 (2.2)	6 (1.9)
Other maternal illness	10 (3.1)	5 (1.6)
Obstetric intervention for fetal reasons	31 (9.6)	33 (10.2)
**Birth weight z score**	-0.4±0.8	-0.4±0.8
**Head circumference (cm) — no.**	215	187
Mean ± SD	23.6±2.2	23.5±2.0
Missing — no.	109	135
**Head circumference z score**	-0.9±1.1	-0.9±1.0
**Baby is one of a multiple pregnancy* — no. (%)**	88 (27.2)	89 (27.6)
Families in the trial (sets of babies from a multiple pregnancy and singletons) — no.	299	292
Sets of babies from a multiple pregnancy in the trial — no.	24	30
**Baby's worst base excess at first hour after birth — no.**	239	252
Mean	-5.6±4.3	-5.6±4.5
Missing no.	85	70
**CRIB II[5] (without temperature) — no.**	239	252
Mean ± SD	11.1±2.6	10.9±2.7
Missing — no.	85	70

* Denotes factor used in the randomization minimization alogorithm

[1] Combines information from seven domains to produce an overall relative measure of deprivation. The domains are income; employment; education; skills and training; health and disability; crime; barriers to housing and services; and living environment.

[2] Doses would usually be 24 hours apart, so < 24 hours before birth suggests only one dose was taken.

[3] Including abnormally implanted placenta.

[4] With or without antepartum hemorrhage.

[5] Clinical Risk Index for Babies.

CONFIDENTIAL

Table S9: Secondary analysis: Restricted analysis excluding infants who received open label medical treatment[1] without meeting the specified criteria

Outcome	Ibuprofen (n = 309)	Placebo (n = 289)	Unadjusted risk ratio (95% CI)	Adjusted risk ratio[2] (95% CI)	p-value
**Death by or moderate/severe BPD at 36 weeks' postmenstrual age — no. (%)**	208 (68.6)	178 (62.5)	1.10 (0.98 to 1.24)	1.09 (0.98 to 1.22)	0.111
Missing — no.	6	4			
**Death by 36 weeks of postmenstrual age — no. (%)**	43 (14.0)	33 (11.5)	1.22 (0.80 to 1.86)	1.20 (0.84 to 1.71)	
Missing — no.	1	1			
**Infants survived up to 36 weeks'— no**	266	256			
**Moderate or severe BPD at 36 weeks of postmenstrual age — no./total no. (%)**	165/260 (63.5)	145/252 (57.5)	1.10 (0.96 to 1.27)	1.10 (0.96 to 1.26)	
Missing — no.	6	4			

[1] Includes medical or surgical management of PDA

[2] Adjusted for size of the PDA, gestational age at birth, age at randomization, sex, center, multiple births, mode of respiratory support at randomisation, and receiving inotropes or not at the time of randomization, and the correlation between siblings from multiple births, where technically possible. Center was treated as a random effect in the models, and all other factors as fixed effects.

Table S10: Additional process outcomes

Outcome	Ibuprofen (n = 324)	Placebo (n = 322)
**Echocardiography not done around 3 weeks of age[1] — no. (%)**	65 (20.1)	60 (18.6)
**Reason: — no./total no. (%)**		
Lack of personnel	25/52 (48.1)	26/48 (54.2)
Baby transferred	10/52 (19.2)	8/48 (16.7)
ECHO done early/late	6/52 (11.5)	3/48 (6.3)
Baby too unwell	3/52 (5.8)	5/48 (10.4)
Not at participating site	4/52 (7.7)	1/48 (2.1)
PDA closed/ no clinical reason	2/52 (3.8)	0/48
Missed in error	1/52 (1.9)	4/48 (8.3)
Other	1/52 (1.9)	1/48 (2.1)
Missing	13	12
**Open-label treatment received without criteria being met, — no. (%)**	15 (4.7)	33 (10.3)
Medical treatment — no./total no. (%)	14/15 (93.3)	28/33 (84.8)
Surgical ligation of PDA — no./total no. (%)	2/15 (13.3)	9/33 (27.3)
Missing — no.	3	3

[1] Within 18-24 days of birth

CONFIDENTIAL

Table S11: PDA ≥ 1.5mm at around 3 weeks by gestational age

Characteristic	Ibuprofen (n = 324)	Placebo (n = 322)
**Total** — no.	**141**	**199**
**Gestational age (weeks)** — no. (%)		
23 to < 24 weeks	21 (14.9)	19 (9.6)
24 to < 25 weeks	36 (25.5)	43 (21.6)
25 to < 26 weeks	34 (24.1)	46 (23.1)
26 to < 27 weeks	24 (17.0)	44 (22.1)
27 to < 28 weeks	17 (12.1)	27 (13.6)
28 to < 29 weeks	9 (6.4)	20 (10.1)
≥ 29 weeks	0	0

Table S12: Post-hoc exploratory analysis: Timing of open-label treatment from randomization

	Ibuprofen (n = 324)	Placebo (n = 322)
**Any open-label treatment — no. (%)**	46 (14.2)	96 (29.8)
**Days from randomization**		
Median (25th, 75th percentiles)	11 (8, 17)	12 (7, 21)
< 1 week after randomization — no./total no. (%)	8/46 (17.4)	20/96 (20.8)
1 to < 2 weeks after randomization — no./total no. (%)	22/46 (47.8)	31/96 (32.3)
2 to < 3 weeks after randomization — no./total no. (%)	7/46 (15.2)	16/96 (16.7)
3 to < 4 weeks after randomization — no./total no. (%)	4/46 (8.7)	10/96 (10.4)
≥ 4 weeks after randomization — no./total no. (%)	5/46 (10.9)	19/96 (19.8)

CONFIDENTIAL

Table S13: Additional secondary outcomes

Outcome	Ibuprofen (n = 324)	Placebo (n = 322)
**Worst stage of ROP[1] in either eye — no. (%)**		
No ROP	142 (43.8)	160 (49.7)
Stage I	58 (17.9)	48 (14.9)
Stage II	66 (20.4)	55 (17.1)
Stage II + disease	2 (0.6)	5 (1.6)
Stage III	31 (9.6)	32 (9.9)
Stage III + disease	22 (6.8)	20 (6.2)
Stage IV	3 (0.9)	1 (0.3)
Stage V	0	1 (0.3)
**AP-ROP[2] in either eye — no. (%)**	10 (3.1)	7 (2.2)
**Timing of significant pulmonary hemorrhage — no./total no. (%)**		
Before trial medication started	2/21 (9.5)	2/17 (11.8)
During trial medication period[3]	1/21 (4.8)	9/17 (52.9)
After trial medication period	18/21 (85.7)	6/17 (35.3)
Missing — no.	3	1
**Diagnosed with acute pulmonary hypertension — no. (%)**	18 (5.6)	20 (6.2)
Missing — **no.**	0	1
**Method of diagnosis**		
Clinically — no. (%)	7 (43.8)	6 (31.6)
Echocardiography — no. (%)	9 (56.3)	13 (68.4)
Missing — no.	2	1
**Treated for acute pulmonary hypertension with pulmonary vasodilator — no. (%)**	17 (5.2)	16 (5.0)
Nitric oxide[4] — no. (%)	11 (73.3)	10 (66.7)
Other[4] — no. (%)	6 (35.3)	10 (52.6)
Missing — no.	0	1
**Severity of BPD[5] at 36 weeks of postmenstrual age (among infants surviving to 36 weeks) — no.**	280	289
No BPD[5] — no./total no. (%)	28/274 (10.2)	37/285 (13.0)
Mild BPD[5] — no./total no. (%)	70/274 (25.5)	79/285 (27.7)
Moderate BPD[5] — no./total no. (%)	61/274 (22.3)	55/285 (19.3)
Severe BPD[5] — no./total no. (%)	115/274 (42.0)	114/285 (40.0)
Missing — no.	6	4
**Hydrocephalus (ventricular index > 4 mm above 97th percentile) — no. (%)**	13 (4.0)	9 (2.8)
**Non-cystic PVL[6] — no. (%)**	2 (0.6)	2 (0.6)
**Other white matter injury — no. (%)**	14 (4.3)	11 (3.4)
**NEC[7] requiring surgery — no. (%)**	19 (5.9)	25 (7.8)
Missing — no.	1	0
**Gastrointestinal bleeding[8] — no. (%)**	9 (2.8)	9 (2.8)
**Spontaneous intestinal perforation — no. (%)**	25 (7.7)	23 (7.1)
Surgical management undertaken — no./total no. (%)	22/25 (88.0)	22/23 (95.7)
**Any open-label treatment[9] — no. (%)**	46 (14.2)	96 (29.8)
**Medical open-label treatment of a symptomatic PDA[10] — no. (%)**	43 (13.3)	82 (25.5)
**Administration of inotropic support — no. (%)**	133 (41.0)	121 (37.6)
**Duration of inotropic support (days) — median (25th, 75th percentiles)**	3 (2 , 6)	3 (2 , 5)

CONFIDENTIAL

Outcome	Ibuprofen (n = 324)	Placebo (n = 322)
(Min to max)	(0 to 28)	(0 to 36)
Missing — no.	0	1
**Diuretics used for management of PDA[10] — no. (%)**	95 (29.3)	125 (38.8)
**Total duration of respiratory support (days) — median (25th, 75th percentiles)**	78 (49 , 106)	78 (51 , 103)
**Invasive ventilation through an endotracheal tube — no. (%)**	305 (94.1)	311 (96.6)
Duration (days), median (25th, 75th percentiles)	10 (3 , 27)	12 (4 , 28)
**Non-invasive respiratory support through nasal CPAP, nasal ventilation, humidified high flow nasal cannula therapy, or low flow oxygen ≥ 1.1 L/min — no. (%)**	289 (89.2)	303 (94.1)
Duration (days) — median (25th, 75th percentiles)	43 (21 , 57)	41 (24 , 56)
**Ambient or low-flow oxygen (< 1.1 L/min) — no. (%)**	254 (78.4)	260 (80.7)
Duration (days) — median (25th, 75th percentiles)	19 (3 , 30)	18 (5 , 31)
**Duration of initial hospitalization (birth to discharge home, in surviving infants) — no.**	265	276
Median (25th, 75th percentiles)	95 (77 , 115)	94 (77 , 117)
Missing — no.	9	6
**Postnatal steroid use for chronic lung disease — no. (%)**	85 (26.3)	82 (25.5)
Missing — no.	1	0
**Head circumference: change in z score between birth and discharge — no.**	146	142
Mean ± SD	-0.0 ± 1.4	0.2 ± 2.1

[1] Retinopathy of prematurity

[2] Aggressive Posterior - retinopathy of prematurity

[3] Date of hemorrhage was on or between the dates of first or last dose. Time of hemorrhage is unknown, so where this occurred on the same day as first or last dose it is not possible to know if this was during the medication period

[4] Categories are not mutually exclusive

[5] Bronchopulmonary dysplasia

[6] Periventricular leukomalacia

[7] Necrotising enterocolitis

[8] Leading to investigation or clinical treatment within 7 days of first dose

[9] Includes medical or surgical management of patent ductus arteriosus

[10] Patent ductus arteriosus

CONFIDENTIAL

Table S14: Safety Events (between the first dose of trial medication and 7 days after the last dose)

Tolerance of ibuprofen treatment within foreseeable SAE reporting range — no. (%)	Ibuprofen (n = 318)[2]	Placebo[1] (n = 317)[2]
Anemia requiring transfusion	192 (60.6)	186 (58.7)
Missing — no.	1	0
Clinically significant intracranial abnormality on cranial ultrasound scan – intracranial hemorrhage or white matter injury	33 (10.4)	20 (6.4)
Missing — no.	2	3
Coagulopathy requiring treatment	31 (9.8)	28 (8.9)
Missing — no.	3	2
Culture proven sepsis	37 (12.3)	36 (12.0)
Missing — no.	18	18
Death	10 (3.1)	6 (1.9)
Fluid retention	36 (11.8)	19 (6.2)
Missing — no.	13	11
Gastrointestinal bleeding	11 (3.5)	11 (3.5)
Hematuria	3 (1.0)	3 (1.0)
Missing — no.	11	11
Hemothorax	2 (0.7)	0
Missing — no.	11	11
High blood creatinine level[3]	64 (20.6)	32 (10.3)
Missing — no.	7	5
Hyperbilirubinemia necessitating exchange transfusion	0	1 (0.3)
Hypotension treated with inotropes	50 (17.1)	37 (12.5)
Missing — no.	25	20
Impaired renal function[4]	71 (22.8)	41 (13.1)
Missing — no.	6	4
Low serum sodium level/hyponatremia[5]	61 (19.3)	77 (24.5)
Missing — no.	2	3
Necrotizing enterocolitis (stage IIA and above)	44 (13.9)	44 (14.0)
Missing — no.	1	3
Neutropenia[6]	30 (10.3)	10 (3.4)
Missing — no.	26	24
Pneumothorax requiring treatment	4 (1.3)	5 (1.6)
Missing — no.	11	12
Pulmonary hypertension requiring treatment with pulmonary vasodilator	5 (1.6)	4 (1.3)
Missing — no.	1	3
Seizures requiring treatment	7 (2.3)	5 (1.6)
Missing — no.	11	11
Significant pulmonary hemorrhage	14 (4.5)	14 (4.5)
Missing — no.	4	3
Spontaneous intestinal perforation	13 (4.1)	17 (5.4)
Thrombocytopenia	23 (7.5)	26 (8.4)
Missing — no.	10	9

SAE denotes serious adverse events

[1] Includes 2 infants who received trial intervention ibuprofen

[2] Based on the safety population defined as: all infants randomised who received at least one dose of the study drug [3] > 100 µmol/L

[4] Urine output < 0.5 mL/kg/hour, and or serum creatinine > 100µmol/L

[5] Sodium < 130 mmol/L

[6] Neutrophils < 1.0 mmol/L

CONFIDENTIAL

Table S15: Unforeseeable serious adverse events

SAE number	Treatment allocation	Center ID	Description	Severity	Causality	Action taken	Outcome
1	Placebo	1	Hyponatremia. Low sodium levels. No Oliguria. No abnormal Renal function results	Moderate	Not related	Discontinued	Resolved
2	Placebo	18	Baby self-extubated. Baby was having long line inserted, ETT dislodged, difficult to intubate, CPR required. Adrenaline and bicarbonate given. Reintubated.	Moderate	Not related	Discontinued	Resolved
3	Ibuprofen	1	Extremely low birth weight baby diagnosed with iatrogenic oesophageal perforation related to gastric tube placement with resulting pneumothorax	Moderate	Not related	None	Resolved
4	Ibuprofen	6	Baby born 24+3 and deteriorated on day 7 of life (developed hypotension, hyperglycemia and a severe metabolic acidosis). Re-intubated and ventilated. Suspected intra-abdominal haemorrhage. Too unstable for transfer to a surgical centre. Progressed to death. Reported as SAE as reason of death unknown. Cause of death since confirmed as intra-abdominal hemorrhage and complications associated with extreme prematurity.	Severe	Not related	None	Fatal
5	Ibuprofen	7	Erosion of the umbilical venous catheter (UVC)through the ductus venosus (initially thought to be a spontaneous intestinal perforation)	Severe	Not related	Discontinued	Resolved
6	Ibuprofen	15	Extravasation of umbilical line, leakage of total parenteral nutrition into peritoneal cavity, coagulopathy and acute renal failure with gross hematuria and metabolic acidosis. Infant made full recovery and was discharged home. Attributed to position of the umbilical venous catheter (UVC) rather than Investigational Medicinal Product (IMP) (confirmed by laparotomy).	Severe	Not related	Discontinued	Resolved
7	Ibuprofen	25	Sudden deterioration, abdominal distention, hypotension and severe metabolic acidosis, suspected NEC/bowel ischemia; progressed to death same day.	Severe	Possibly related	Discontinued	Fatal
**Suspected Unexpected Serious Adverse Reaction (SUSAR)**							
8	Ibuprofen	3	Baby had falling hemoglobin, poor perfusion and was pale, with increasing abdominal distention. Not resolving after blood transfusions. Abdominal ultrasound performed	Severe	Possibly related	Discontinued	Fatal

SAE number	Treatment allocation	Centre ID	Description	Severity	Causality	Action taken	Outcome
			suggesting a 4cm subcapsular hematoma, no evidence of peritoneal collection, looks localized.<br>Baby was managed conservatively thereafter with evidence of a reduction in size of the hematoma. Developed progressive cardiorespiratory failure managed with high frequency oscillatory ventilation (HFOV), nitric oxide and inotropic support. Developed a Coagulase-negative staphylococci (CONS) bloodstream infection and PIE changes on chest x-ray. Treated with dexamethasone with a partial response. ECHO showed a non-specific pattern of diffuse echogenic lesions scattered throughout the endo/myocardium of both ventricles. Cranial US scans were normal. Despite maximal intensive care support, continued to deteriorate.<br>The cause of death was certified as severe pulmonary interstitial emphysema (PIE), extreme prematurity, acute renal failure and *Staphylococcal hemolyticus* sepsis.				

Table S16: Representativeness of Study Participants

**Category**	**Patent Ductus Arteriosus (PDA)**
Disease, problem, or condition under investigation	Selective early medical treatment of patent ductus arteriosus with Ibuprofen
Special considerations related to:	
Sex and gender	Patent ductus arteriosus in premature babies equally affects male and female infants
Age	Patent ductus arteriosus is a common morbidity in extreme premature babies born at less than 29 weeks of gestation. The prevalence of patent ductus arteriosus is inversely proportional to the gestational age of the infant.
Race or ethnic group	Patent ductus arteriosus affects premature babies irrespective of ethnicity and race.
Geography	There is no reported geographical variation with regards to the prevalence of patent ductus arteriosus in premature infants.
Other considerations	Severity of respiratory distress syndrome and the need for respiratory support for respiratory distress syndrome are associated with the prevalence of patent ductus arteriosus. Persistence of patent ductus arteriosus has been reported to be associated with mortality and other complications of prematurity throughout the world and is more common in infants born below 26 weeks of gestation.
Overall representativeness of this trial	The participants in the trial demonstrate the expected ratio of males to females. The ethnicity breakdown is also representative of the UK population with 74% of infants born to White mothers. As extreme premature infants were screened for the presence of patent ductus arteriosus for inclusion in the trial, all infants required respiratory support at randomization, reflecting underlying respiratory distress syndrome.

References

1. Schmidt B, Davis P, Moddemann D, Ohlsson A, Roberts RS, Saigal S, Solimano A, Vincer M, Wright LL; Trial of Indomethacin Prophylaxis in Preterms Investigators. Long-term effects of indomethacin prophylaxis in extremely-low-birth-weight infants. N Engl J Med. 2001;344:1966-72. doi: 10.1056/NEJM200106283442602. PMID: 11430325.

2. Schmidt B, Roberts RS, Fanaroff A, Davis P, Kirpalani HM, Nwaesei C, Vincer M; TIPP Investigators. Indomethacin prophylaxis, patent ductus arteriosus, and the risk of bronchopulmonary dysplasia: further analyses from the Trial of Indomethacin Prophylaxis in Preterms (TIPP). J Pediatr. 2006;148:730-734. doi: 10.1016/j.jpeds.2006.01.047. PMID: 16769377.

3. Neonatal Survey Database from the Trent Region 2010; https://www.le.ac.uk/departments/health-sciences/research/timms/projects/tns. Accessed 8 Dec 2020.

4. Noori S. Patent ductus arteriosus in the preterm infant: to treat or not to treat? J Perinatol. 2010;30 Suppl:S31-7. doi: 10.1038/jp.2010.97. PMID: 20877405.
